# Divergent Avian Influenza H10 Viruses from Sympatric Waterbird Species in Italy: Zoonotic Potential Assessment by Molecular Markers

**DOI:** 10.3390/microorganisms13112575

**Published:** 2025-11-12

**Authors:** Marzia Facchini, Maria Alessandra De Marco, Sara Piacentini, Angela Di Martino, Cesare Ernesto Maria Gruber, Claudia Cotti, Giuseppina Di Mario, Laura Calzoletti, Concetta Fabiani, Mauro Delogu, Anna Teresa Palamara, Paola Stefanelli, Simona Puzelli

**Affiliations:** 1 Department of Infectious Diseases, Istituto Superiore di Sanità (ISS), 00161 Rome, Italy; sara.piacentini@iss.it (S.P.); angela.dimartino@iss.it (A.D.M.); giuseppina.dimario@iss.it (G.D.M.); laura.calzoletti@iss.it (L.C.); concetta.fabiani@iss.it (C.F.); annateresa.palamara@iss.it (A.T.P.); paola.stefanelli@iss.it (P.S.); simona.puzelli@iss.it (S.P.); 2 Wildlife Service, Institute for Environmental Protection and Research (ISPRA), 40064 Ozzano dell’Emilia, Italy; mariaalessandra.demarco@isprambiente.it; 3 Laboratory of Virology, National Institute for Infectious Diseases ‘Lazzaro Spallanzani’—IRCCS, 00149 Rome, Italy; cesare.gruber@inmi.it; 4 Wildlife and Exotic Animal Service, Department of Veterinary Medical Sciences, University of Bologna, 40064 Ozzano dell’Emilia, Italy; claudia.cotti@unibo.it (C.C.); mauro.delogu@unibo.it (M.D.)

**Keywords:** avian influenza virus, H10, molecular markers, divergent H10 viruses, *Anas platyrhynchos*, *Fulica atra*, zoonotic potential, sympatric waterbird species, Mallard, Eurasian coot

## Abstract

Avian influenza viruses (AIVs) of H10 subtype are able to circulate in domestic and wild bird populations but can also spill over and adapt to mammals, posing a continuous risk to biodiversity conservation, veterinary health, and public health. In the present study, we assessed the zoonotic potential of nine H10 AIVs isolated from waterbirds during surveillance and research studies carried out in Italy between 1994 and 2007. Overall, six H10NX strains from wild mallards (n. 1 H10N2, n. 5 H10N7), one H10N7 strain from domestic mallards, and two H10N8 strains from Eurasian coots were sequenced by next-generation sequencing (NGS). HA phylogenetic analysis indicated a marked divergence between viruses from these two sympatric waterbird species and showed a close relationship between three H10N7 strains from wild mallard and one H10N7 isolate of domestic origin. Sequence analysis revealed the presence of several molecular markers, associated with increased zoonotic potential, including the PB2-A588V mutation found in the Eurasian coot H10N8 viruses and previously linked to mammalian adaptation in H10 strains. Molecular analysis also showed that all H10 viruses were susceptible to the major approved classes of influenza antivirals (inhibitors of neuraminidase, matrix-2, and polymerase acid protein). Moreover, phenotypic assay confirmed their susceptibility to oseltamivir and zanamivir drugs. From an ecological perspective, we found that different H10 gene pools seem to be harboured in different waterbird species sharing the same environment; additionally, a bidirectional transmission of H10 mallard isolates occurred between natural and anthropic ecosystems. Overall, our findings account for the need of continuous monitoring of AIVs belonging to the H10 subtype.

## 1. Introduction

Wild waterbirds—the major reservoir of the influenza A virus gene pool—harbour all the haemagglutinin (HA) and neuraminidase (NA) subtypes of avian influenza viruses (AIVs) in their natural habitats, including the sixteen HA (H1 to H16) and the nine NA (N1 to N9) known so far [1,2]. All the sixteen HA subtypes circulate in wild waterbirds as low pathogenicity (LP) strains, and only the H5 and H7 subtypes can occasionally mutate in poultry into highly pathogenic (HP) AIVs. As shown by the recent global spread of H5 HPAIVs of clade 2.3.4.4, virus–host–environment ecological interface can enable interspecies transmission of AIVs between wild waterbirds and other avian and/or mammalian species, thus generating potential risks for animal and human health as well as biodiversity conservation [3,4,5]. Consequently, the meaning of “notifiable disease”—defined as H5 and H7 HPAIV infections in poultry and birds other than poultry, including wild birds—has been extended by the World Organisation for Animal Health to both LPAIV infections showing a sudden and unexpected increase in virulence in poultry and LPAIV infections of domestic and captive wild birds with proven natural transmission to humans associated with severe consequences [6].

Several HA subtypes of LPAIVs can affect poultry production and human health by zoonotic transmission and possible reassortment events with other influenza A viruses [7]. In recent years, AIVs belonging to H10 subtype have progressively posed a threat to public health due to their capability to infect and adapt to mammals, including humans. The first H10 AIV was isolated from a chicken in Germany in 1949 (H10N7) [8]. Since then, H10NX viruses and/or H10 seropositivity were detected not only in domestic and wild birds but also in domestic and wild mammalian species (mink, harbour seal, pig, raccoon, feral dog) worldwide [8,9,10]. Moreover, sporadic human infections caused by H10 AIV subtype were reported in Egypt (2004), Australia (2010), and China (2013, 2014, 2021, 2022, 2024, and 2025) [11,12,13,14,15,16]. In this context, it is becoming increasingly important to control the spread of H10NX AIVs by implementing virological and serological monitoring systems in both animal and human populations.

In this study, we conducted a retrospective analysis of a group of H10 subtype viruses isolated in Italy between 1994 and 2007 from two sympatric wild species of waterbirds—mallard (*Anas platyrhynchos*) and Eurasian coot (*Fulica atra*)—and from domestic mallards (*Anas platyrhynchos* domestic form). We have sequenced the complete genomes of nine H10 AIVs in order to achieve the following: (i) analyse the presence of known molecular markers associated with pathogenicity and host adaptation; (ii) investigate their evolutionary relationships with other Eurasian human and avian H10 viruses and determine whether, and to what extent, Italian H10 strains isolated from two sympatric avian species were related; and (iii) analyse the presence of mutations associated with adamantanes resistance and reduced susceptibility to neuraminidase inhibitors (NAIs) and polymerase acidic inhibitors (PAIs). Furthermore, the susceptibility of H10 isolates to the neuraminidase inhibitors oseltamivir, and zanamivir was tested by phenotypic assays.

## 2. Materials and Methods

### 2.1. Sample Collection and Virus Isolation

The nine H10 AIV analysed (Table 1) were previously isolated from wild and domestic waterbirds during surveillance and research studies carried out in Italy between 1994 and 2007 [17,18,19].

**Figure 1:**
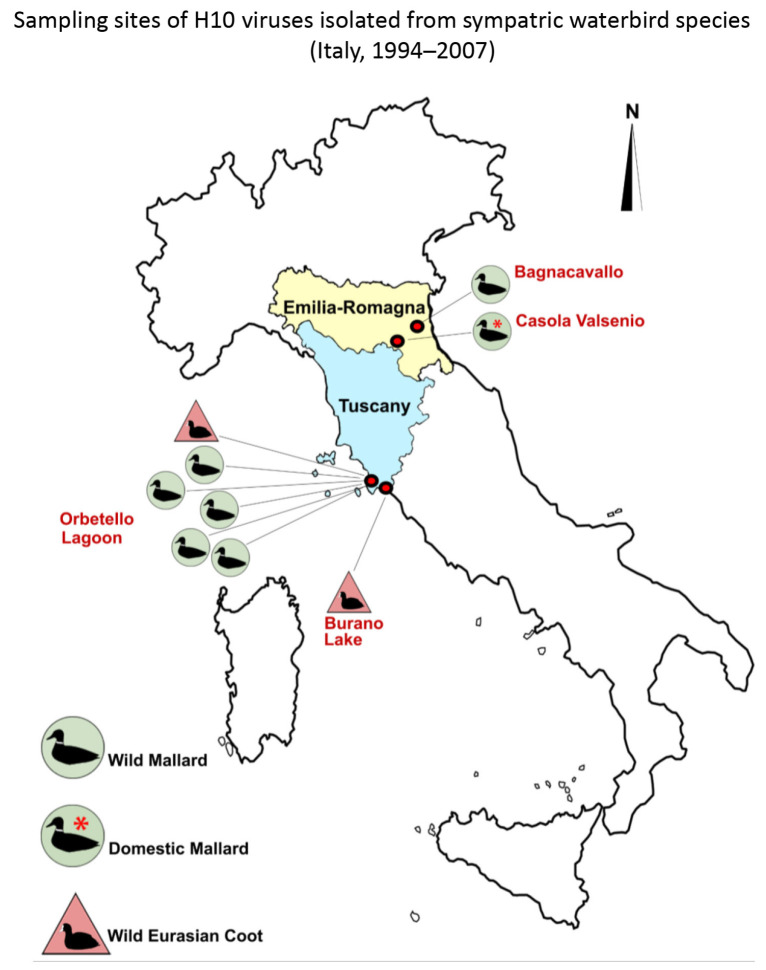
Map showing geographic areas and waterbird species sampled in the Tuscany and Emilia-Romagna regions. Sampling sites and related H10 isolate numbers were as follows: Orbetello Lagoon (n. 6), Burano Lake (n. 1), Bagnacavallo (n. 1), and Casola Valsenio (n. 1).

A map showing H10 sampling sites and the waterbird species sources of these isolates is reported in Figure 1.

Briefly, the following procedures were performed in two different geographic areas:

(i) During bird-ringing activities carried out in the Laguna di Orbetello Oasis and Lago di Burano World Wildlife Fund Oasis—protected wetlands placed on the west coast of Central Italy in the Tuscany region, about 140 km north of Rome—seven cloacal swabs were collected between 1994 and 2006 from two Eurasian coots (*Fulica atra*) and five mallards (*Anas platyrhynchos*). These samples were individually processed and inoculated according to standard procedures in specific pathogen-free (SPF)-embryonated chicken eggs for virus isolation [20], followed by influenza A virus detection and characterisation by hemagglutination (HA) assay [21], enzyme-linked immunosorbent assay (ELISA) [22], RT-PCR [23], and finally subtyped by hemagglutination inhibition (HI) and neuraminidase inhibition (NI) tests [21].
(ii) During the AIV Surveillance Plan implemented in the Emilia-Romagna Region of northern Italy, one pool of 10 cloacal swabs obtained from a group of 2000 free-range mallards reared in an open-air breeding farm (*Anas platyrhynchos* domestic farm), and one cloacal swab obtained from an injured wild mallard (recovered in the upper valley of the Senio River), were processed to be inoculated into SPF-embryonated chicken eggs [20]. The harvested allantoic fluids were tested by the HA assay [21] and an ELISA specific for the detection of influenza A virus nucleoprotein [22]. Allantoic fluids that tested positive by both HA and ELISA were sent to the Italian National Reference Laboratory for Avian Influenza and Newcastle Disease (Legnaro, PD) for antigenic subtype and pathotype characterisation.

### 2.2. Sample Processing and Testing

#### 2.2.1. RNA Extraction and Whole Genome Sequencing

Viral RNAs were extracted from isolates using the QIAamp Viral RNA extraction kit (Qiagen, Hilden, Germany). The entire genome of each H10 virus was amplified by multisegment reverse transcription-PCR with MBTuni-12 and MBTuni-13 primers using the SuperSript III one-step RT-PCR System with Platinum Taq DNA Polymerase (Invitrogen, Waltham, MA, USA), according to the protocol of Zhou et al. [24].

Next-generation sequencing was performed using the Illumina MiSeq Next Generation platform (Illumina, San Diego, CA, USA).

Raw reads were filtered using the Trimmomatic tool v.0.36 [25], as follows: dynamic trimming was adopted to remove read fragments of 15 nt in length and with a mean Phred quality score <30; trimmed reads shorter than 100 nt were discarded. All reads were examined for mean quality, length, GC, and adapter content before and after trimming process with FastQC software v0.11.5 (Babraham Institute, Cambridge, UK). Whole genome sequence assembly, variant calling, and phasing were performed using the Iterative Refinement Meta-Assembler (IRMA) pipeline v.1.2.0 [26]. Complete H10 segment sequences were aligned using the MAFFT software v7.505 [27] and manually checked with Geneious Prime v.2020.2.5 (Dotmatics, Boston, MA, USA). The complete genome sequences were submitted to GISAID (“global initiative on sharing avian flu data”, https://www.gisaid.org, accessed on 28 October 2025) EpiFlu database. Sequence accession numbers in GISAID were as follows: EPI_ISL_20096817 for A/Eurasian Coot/Italy/125/1994; EPI_ISL_20096818 for A/Eurasian Coot/Italy/114/1995; EPI_ISL_20096819 for A/Mallard/Italy/90/2002; EPI_ISL_20096820 for A/Mallard/Italy/166998/2005; EPI_ISL_20096822 for A/Mallard/Italy/Eco-634/2005; EPI_ISL_20096945 for A/Mallard/Italy/Eco-7/2006; EPI_ISL_20097047 for A/Mallard/Italy/Eco-33/2006; EPI_ISL_20097048 for A/Mallard/Italy/Eco-360/2006; and EPI_ISL_20097050 for A/Mallard/Italy/195376/2007.

#### 2.2.2. Sequence and Phylogenetic Analyses

The sequences analysed in this study were aligned and compared with those available from GISAID, edited, and analysed using the CLUSTAL X programme v.1.4 [28] and BioEdit 7.05 version [29]. The Megalign programme, included in the DNASTAR Lasergene v.15 software (Lasergene software, DNASTAR Inc., Madison, WI, USA), was also used to analyse nucleotide sequence identity.

For HA phylogenetic analysis, sequences downloaded from BLAST (accessed on 24 June 2025) results obtained from GISAID were used, together with representative sequences from H10NX Eurasian viruses collected from 1970 to 2024, including sequences from human H10 viruses isolated in China and from other mammalian species. Some HA sequences of the North American lineage were also added. The HA phylogenetic tree was created using the maximum likelihood method with IQ-Tree v.16.12 and ModelFinder software v.1.0 to select the best tree model [30], with 5000 bootstrap replicates.

#### 2.2.3. Neuraminidase Inhibitors and NA Inhibition Test

Oseltamivir carboxylate (GS4071) and zanamivir compounds were kindly provided by Roche and GlaxoSmithKline, respectively.

A fluorescent enzyme inhibition assay was used in the present study to test the H10NX AIVs susceptibility to NI drugs and to determine the inhibitory drug concentration (IC_50_) [31]. Briefly, H10NX were screened for susceptibility to NAIs using the 2′-(4-methylumbelliferyl)-a-D-N-acetylneuraminic acid, sodium salt hydrate (MUNANA: Sigma-Aldrich, St Louis, MO) as the substrate. Each isolate was initially titrated in black 96-well flat bottom plates in order to standardise virus input. After titrating NA activities, the inhibition assay was performed by preincubating 10 μL of drug and 10 μL of diluted virus for 30 min at 37 °C. Then, 100 μM of working MUNANA solution was added to each well, and plates were incubated for 1 h at 37 °C. The reaction was stopped, and fluorescence was measured in a fluorometer with an excitation wavelength of 355 nm and an emission wavelength of 460 nm.

Criteria recommended by the World Health Organisation Antiviral Working Group, based on the fold change in IC_50_ compared to a susceptible virus, were used to define the susceptibility of H10NX strains to NAIs. According to these criteria, Influenza A isolates showed the following: normal inhibition (<10-fold), reduced inhibition (10- to 100-fold), and highly reduced inhibition (>100-fold) [32].

A/Victoria/4897/2022 (H1N1pdm09) and A/Darwin/9/2021 (H3N2) strains were included in the assay as wild-type reference viruses. An oseltamivir-resistant H1N1pdm09 isolate with the NA-H275Y amino acid substitution was also included in the test. IC_50_ values (the drug concentration that inhibited 50% of the NA activity) were representative of two independent assays.

## 3. Results

### 3.1. HA Phylogenetic Analysis

**Figure 2:**
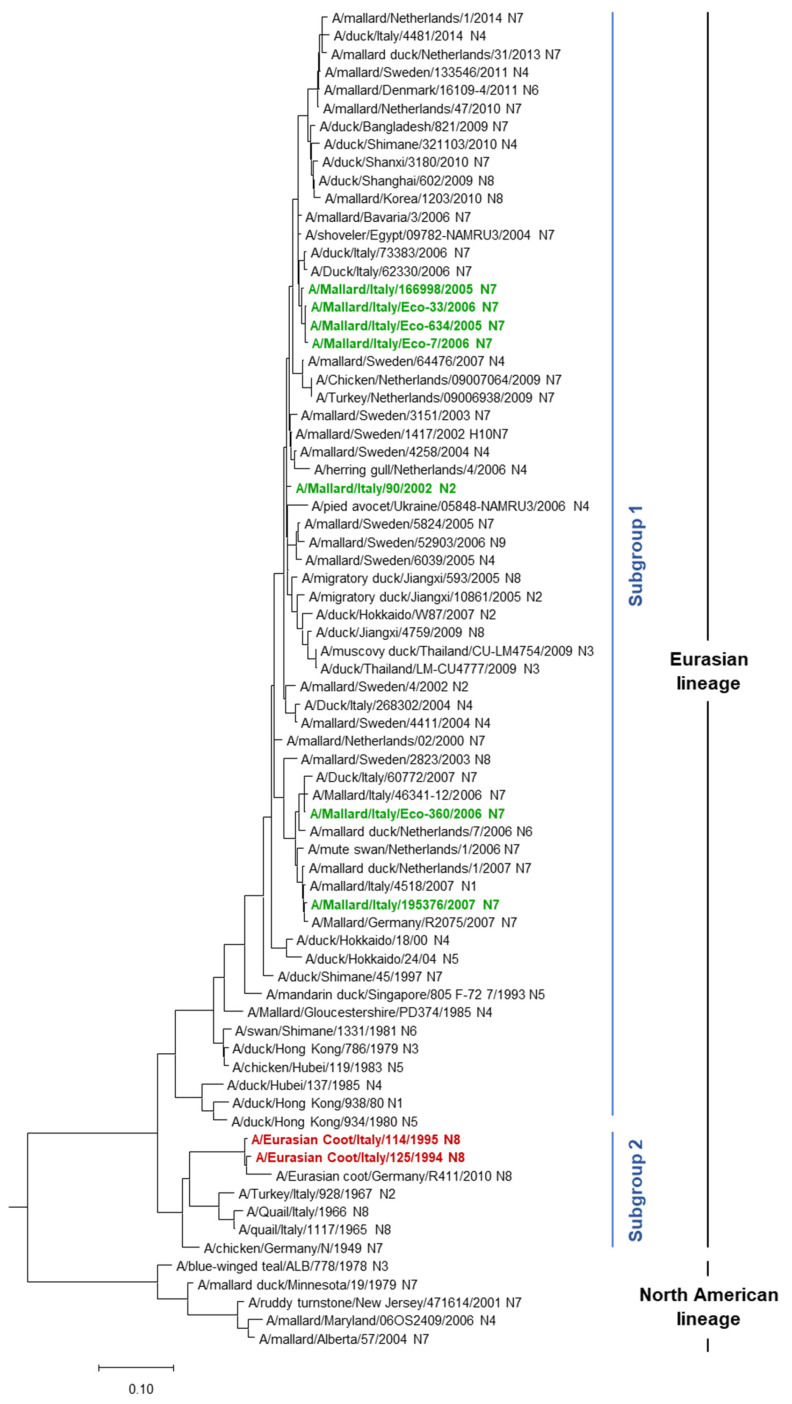
Phylogenetic tree of H10 genes of Italian AIVs under study. AIVs isolated from mallards and Eurasian coots are represented in green and red, respectively. The phylogenetic tree was generated using the maximum likelihood algorithm with 5000 bootstrap replicates.

Phylogenetic analysis of the HA genes showed that all the Italian H10 strains belonged to the Eurasian lineage and fell into two subgroups (Figure 2).

In particular, subgroup 1 contained all seven strains that were isolated from the mallards, while subgroup 2 contained the coot-origin viruses. In subgroup 1, the H10 isolates were grouped together with viruses that were circulating mainly in Europe, predominantly in mallards and other duck species, during the years 2000–2007. More specifically, the two H10N7 strains A/Mallard/Italy/Eco-360/2006 and A/Mallard/Italy/195376/2007 were closely related to viruses that were also circulating in Italy at that time, A/Mallard/Italy/46341-12/2006 (H10N7) and A/Mallard/Italy/4518/2007 (H10N1), respectively, with A/Mallard/Italy/195376/2007 isolate sharing the highest nucleotide identity (99%—BLAST result, accessed on 24 June 2025) with a virus isolated in Germany (H10N7 A/Mallard/Germany/R2075/2007) (Table 2).

The other four H10N7 isolates, A/Mallard/Italy/166998/2005, A/Mallard/Italy/Eco-634/2005, A/Mallard/Italy/Eco-33/2006, and A/Mallard/Italy/Eco-7/2006 clustered together (Figure 2), and all of them showed the highest nucleotide identity with A/H10N7 A/Shoveler/Egypt/09781-NAMRU3/2004 (98–99%—BLAST result) (Table 2). The H10N2 A/Mallard/Italy/90/2002 isolate, instead, predominantly showed a close relationship with viruses that were circulating in Sweden and shared the highest identity with H10N7 A/Mallard/Sweden/1417/2002 (98%—BLAST result).

In subgroup 2, the two H10N8 coot-origin strains, A/Eurasian Coot/Italy/125/1994 and A/Eurasian Coot/Italy/114/1995, clustered together with H10N8 A/Eurasian coot/Germany/R411/2010 virus, shared 96% of nucleotide identity (BLAST result).

**Figure A1:**
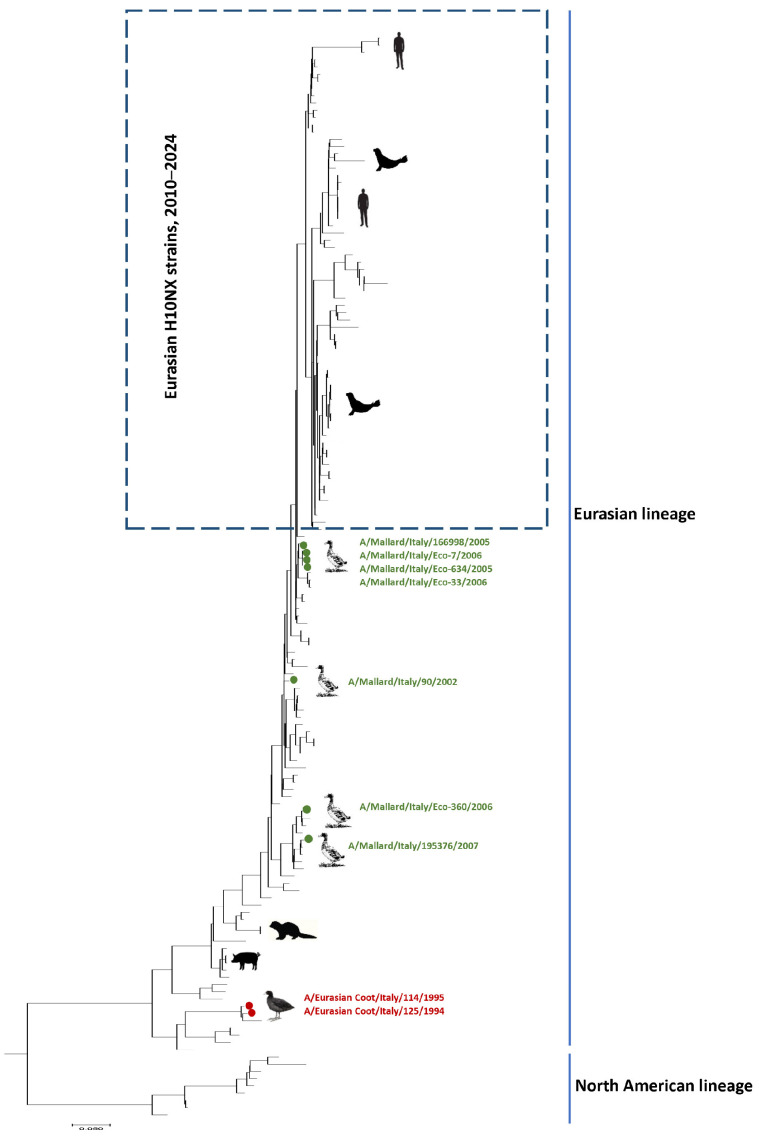
Phylogenetic tree of the HA genes of the H10 viruses. AIVs isolated from mallards and Eurasian coots are represented in green and red, respectively. Recent H10NX viruses of Eurasian lineage are included in the dotted box. The phylogenetic tree was generated using the maximum likelihood algorithm with 5000 bootstrap replicates. Names of H10NX strains used in the tree are shown in Supplementary Materials (Figure S9).

As shown in the Appendix A and Supplementary Materials (Figure A1 and Figure S9), none of our H10 under study had a close relationship with the more recent human H10N8 (A/Jiangxi-Donghu/346/2013, A/Jiangxi/09037/2014) and H10N3 (A/Jiangsu/428/2021, A/Yunnan/0110/2024, A/Guangxi/01591/2024) isolates from China. In addition, they did not show any close relationship with the H10 strains that were isolated in other mammalian species including minks (H10N4, 1984), pigs (H10N5, 2008), and harbour seals (H10N7, 2014, 2015, and 2021) [8,33]. We also found that none of our H10 were closely related to more recent Eurasian H10 AIVs identified over the last few years (Figure A1).

### 3.2. Molecular Characterisation of the H10 AIVs

#### 3.2.1. Nucleotide Identity

Regarding the HA gene, at the nucleotide level (Figure S1), a low degree of similarity was observed between the H10 strains isolated from Eurasian coots and those from mallards. Particularly, the HA similarity percentage observed between the two viruses A/Eurasian Coot/Italy/125/1994 and A/Eurasian Coot/Italy/114/1995 was 99% and this percentage was found to be lower when these strains were compared with those isolated from mallards that ranged from 83.6 to 84.9% (Figure S1). Among the seven H10 mallard-origin strains, the HA nucleotide similarity percentage varied from 93.2 to 99.9%, with the highest values (99.2–99.9%) observed among A/Mallard/Italy/166998/2005, A/Mallard/Italy/Eco-634/2005, A/Mallard/Italy/Eco-7/2006, and A/Mallard/Italy/Eco-33/2006 (Figure S1). Concerning the NA genes, N8 neuraminidase of A/Eurasian Coot/Italy/125/1994 and A/Eurasian Coot/Italy/114/1995 were 99.2% similar. Additionally, NA nucleotide similarity percentages among the six H10N7 viruses ranged from 92.4 to 99.9% (Figure S2). Examination of the sequence of the six internal protein genes of the two virus groups from Eurasian coots and mallards revealed comparable results to those observed for the HA genes. In this regard, the two Eurasian coot-origin strains shared high similarity percentages from 99.1% to 99.7% across all segments, but these values decreased when these isolates were compared with viruses from mallards, with the exception of the MP and NS genes, for which higher values were found (Figures S3–S8). More in detail, the ranges of similarity percentages were as follows: 88.5 to 89.9% for NP gene (Figure S3); 89.4 to 90.6% for PA gene (Figure S4); 90.7 to 91.4% for PB1 gene (Figure S5); 87.7 to 88.6% for PB2 gene (Figure S6); 95.6 to 97% for MP gene (Figure S7); and 72 to 92.5% for NS gene (Figure S8).

The nucleotide similarity values of internal genes ranged from 92.5 to 100% in most viruses isolated from the mallards. Noteworthy, one virus, A/Mallard/Italy/195376/2007, showed very low similarity values for the NS gene (71.7–72%) when compared with the other H10 mallard-origin strains under study.

#### 3.2.2. HA Gene

Haemagglutinin amino acid sequence analysis of the seven H10 AIVs isolated from mallards showed the presence of the PEIMQGR/GLF motif at the cleavage site, typically associated with an LP (low pathogenicity) phenotype. Interestingly, two viruses, A/Eurasian Coot/Italy/125/1994 and A/Eurasian Coot/Italy/114/1995, presented a different cleavage pattern, PEVVQGR/GLF, usually observed in the H10 viruses belonging to the North American lineage [10,34] (Table 3).

No mutations in the 226 and 228 amino acid positions (H3 numbering) were observed in residues that define the receptor-binding site (RBS) of all H10 viruses, indicating their ability to preferentially bind to the avian Neu5Aca2,3-Gal receptor determinants [4]. However, the S221P amino acid change (S231P in H10 numbering), which may potentially increase the virus binding to the human Neu5Aca2,6-Gal receptor, was found [35,36]. (Table 4).

Finally, molecular markers in HA associated with altered viral fitness and transmissibility of AIVs reported in Table S1 [36,37,38,39,40,41,42,43,44,45,46,47,48,49,50,51,52,53,54,55,56,57,58,59,60,61,62,63,64,65,66,67,68,69,70,71,72,73,74,75,76,77,78,79,80,81] were not found in the H10 isolates under study, even though the K393E (H3 numbering) aminoacidic substitution, known to be related to increased pH of fusion, decreased HA stability, and reduced virulence in mice for the H7N9 subtype [36,37], was detected in all H10 viruses under study.

#### 3.2.3. NA Gene

Analysis of NA amino acids forming the catalytic site involved in the release of the progeny virions from the surface of infected cells (R118, D151, R152, R224, E276, R292, R371, and Y406—N2 numbering) did not reveal any changes in the viruses analysed. No mutations were identified in the eleven amino acids of the framework region that are involved in the stabilisation of the NA active site (E119, R156, W178, S179, D198, I222, E227, H274, E277, N294, and E425—N2 numbering) [82], except for a D198N aminoacidic substitution found in the N7 of all analysed H10N7 viruses. It was not possible to verify the 406 and 425 amino acid positions for only one isolate, A/Mallard/Italy/90/2002, because of its shorter N2 sequence.

Additionally, no molecular markers reported in Table S2 and known to be associated with reduced susceptibility to neuraminidase inhibitors, oseltamivir, and zanamivir [83,84,85,86,87,88,89,90,91,92,93] were found in the N8, N7, and N2 amino acid sequences of our H10 isolates. Also, no deletions related to increased transmission in mammalian cells [94] or those reported in Table S3 and associated with enhanced virulence in mice [36,95,96,97,98,99,100] were observed.

#### 3.2.4. Internal Protein Genes

None of the H10 isolates under study harboured the amino acid changes that are more frequently associated with the adaptation of AIVs to mammalian species in the PB2 (E627K, D701N) and PB1-F2 (N66S) genes. In addition, eight out of the nine H10 viruses had the four C-terminal amino acids motif ESEV in the NS1 protein, typical of avian influenza viruses [101], whereas the isolate A/Eurasian Coot/Italy/114/1995 showed the presence of a stop codon in it (ES*V).

The screening of M2 mutations did not reveal the presence of substitutions known to confer resistance to adamantanes [102]. Concerning susceptibility to the polymerase acidic inhibitor baloxavir marboxil, the PA-L28P genetic change—the PA marker associated with reduced susceptibility to this antiviral in seasonal human influenza A(H3N2) viruses—was detected [103], although the presence of a proline in this position (P28) could represent a conserved characteristic of AIVs [104]. Interestingly, the analysis of amino acid substitution in internal proteins of our H10 isolates revealed several mutations previously reported to be linked to an increase in the zoonotic potential in different avian influenza subtypes [36]. As shown in Table A1 [105,106,107,108,109,110,111,112,113,114,115,116,117,118,119] and in Table S4, the following mutations were found in our H10 isolates: in PB2, R340K, K389R, A588V, V598T, L89V + G309D, and L89V + G309D + T339K + R477G + I495V + K627E + A676T [105,106,107]; in PB1, D3V and D622G [108,109]; in PA, S37A, N383D, and N409S [110,111]; in NP, M105V, I109T, and A184K [112,113]; in M1, N30D, I43M, and T215A [114,115]; in NS1, P42S, C138F, V149A, L103F + I106M, and K55E + K66E + C138F [116,117,118,119]. Overall, 18 mutations and 4 motifs were found in the internal genes of H10 isolates. The distribution of mutations/motifs by gene, strain origin, and sample collection date is shown in Table 5.

In detail, all the mutations/motifs that we detected in PB2 (4/2), PB1 (2/0), PA (3/0), M1 (3/0), and NS1 (3/2) were found in the two Eurasian coot isolates, whereas only one of the three NP-mutation (A184K) was found in these H10N8 viruses. The seven H10 AIVs isolated from mallards had all the mutations/motifs that we detected in PB1, PA, and M1, whereas two or three mutations and two motifs were found in the PB2 gene. All NS1 mutations/motifs (3/2) were found in six of seven mallard strains, whereas only two mutations were observed in the 31 July 2007 isolate. Finally, the distribution of NP mutations in mallard isolates was as follows: A184K found in the unique H10N2 and in all the H10N7 strains; M105V found in all six H10N7 strains; and I109T only found in the H10N7 virus sampled on 24 January 2006 (see also Table S4 and Table A1).

### 3.3. Antiviral Susceptibility by Phenotypic Assay

H10NX AIVs were also examined for their susceptibility to the NA inhibitors oseltamivir and zanamivir by phenotypic assay. All isolates tested, with the exception of A/Mallard/Italy/166998/2005, for which it was not possible to perform the assay due to its low neuraminidase activity, were sensitive to both drugs. These strains showed mean IC_50_ (mean 50% inhibitory concentration) values ranging from 0.3 to 3.4 nM for oseltamivir and from 0.7 to 1.3 nM for zanamivir (Table 6). Furthermore, all viruses tested exhibited less than a 10-fold increase in oseltamivir and zanamivir IC_50_ compared with the mean of reference wild-type viruses used in the assay and with the IC_50_ values previously reported in the literature for N8, N2, and N7 susceptible viruses [84,85,120], indicating that they were susceptible to both drugs.

## 4. Discussion

In recent decades, H10 subtype AIVs such as H10N3, H10N4, H10N5, H10N7, and H10N8 have crossed species barriers, confirming their ability to infect several mammalian species, including domestic and wild animals as well as humans, thus posing a threat to public health, veterinary health, and biodiversity conservation [3,4,5,11,12,13,14,15,16,121,122]. Furthermore, serological evidence of H10 infections has been reported in wild and domestic mammals [8,9,10] as well as in occupationally exposed workers [123]. Given the occurrence of these events, it is now becoming increasingly important to keep these avian influenza strains under control by monitoring their circulation in both wild and domestic birds, as well as in mammals, including humans.

Our retrospective study provides molecular and phylogenetic data of H10 AIVs isolated in Italy between 1994 and 2007 from wild mallards (*Anas platyrhynchos*), Eurasian coots (*Fulica atra*), and from reared mallards (*Anas platyrhynchos* domestic form) that were exposed to avian influenza through natural wetland habitats and open-air free-range farms, respectively.

According to the census of wintering waterbirds [124], in Italy, large flocks of these two avian species overwinter in Italian wetlands, and during the study period, the mallard was the most widespread and abundant anatid species, showing an increasing trend (242,022 vs. 72,383 individuals, respectively, in the 2006–2010 and 1991–1995 periods). Similarly, the Eurasian coot was found to be the most abundant species in Italy (263,976 vs. 215,010 individuals, respectively, in the 2006–2010 and 1991–1995 periods). In this context, both wintering avian species were primarily represented by migratory contingents coming from breeding sites mainly represented by wetlands in Central and North-Eastern Europe and, to a lesser extent, by sedentary populations (accounting for 10,000–20,000 pairs and 8000–12,000 pairs, respectively, estimated for mallards and Eurasian coots) [125,126]. It should be noted that the above mentioned domestic mallards—belonging to the taxon artificially selected for food, ornamental, and hunting uses—could occur in the wild because of accidental or voluntary releases (e.g., 5149 individuals of this taxon were counted in Italian wetlands during the 2006–2010 period) [124]. This anthropogenic interface could influence AIV ecology, as reported below.

HA phylogenetic analysis of the nine H10NX viruses under study indicated that all these strains belonged to the Eurasian lineage. None of the H10 showed a close relationship with Eurasian human and avian H10 AIVs identified in more recent years, as well as no observed relationship with H10 strains isolated in other mammalian species including minks, pigs, and harbour seals [8,33]. Interestingly, the H10 viruses isolated from mallards and coots fell into different subgroups, subgroup 1 and subgroup 2, respectively (Figure 2). In subgroup 1, the mallard isolates analysed clustered with H10 viruses almost exclusively circulating in Anseriformes in Europe during the years 2000–2007. Among these, four H10N7 grouped together as follows: three strains from wild mallards captured in Central Italy (A/Mallard/Italy/Eco-634/2005, A/Mallard/Italy/Eco-33/2006, and A/Mallard/Italy/Eco-7/2006) and one (A/Mallard/Italy/166998/2005) from domestic mallards reared in Northern Italy in a lowland area at high risk for AIV introduction into bird farms [19]. Interestingly, the HA genes of these three H10N7 viruses isolated from wild mallards in December 2005 and January 2006 were closely related to that of the H10N7 strain isolated from domestic mallards in July 2005. This result seems to confirm that free-range farms of *Anas platyrhynchos* domestic type can represent a wildfowl–poultry interface, allowing bidirectional transmission of AIVs between outdoor-housed ducks and wild waterfowl [19]. Moreover, the two H10N7 isolates of wild origin, A/Mallard/Italy/Eco-360/2006 and A/Mallard/Italy/195376/2007, obtained in Central and Northern Italy, respectively, were closely related to other strains also co-circulating in mallards in Italy, with A/Mallard/Italy/195376/2007 sharing the highest nucleotide identity (99%) with the H10N7 A/Mallard/Germany/R2075/2007 strain. In subgroup 2, A/Eurasian Coot/Italy/125/1994 and A/Eurasian Coot/Italy/114/1995 shared a relationship (96% HA nucleotide identity) only with A/Eurasian coot/Germany/R411/2010, an H10N8 strain previously isolated in Germany in 2010. Within the same subgroup, these coot isolates seem to share a common origin with the old H10NX strains, including the first reported H10N7 (A/chicken/Germany/N/1949) and three H10 viruses (A/quail/Italy/1117/1965-H10N8, A/Quail/Italy/1966-H10N8), A/Turkey/928/1967-H10N2) isolated in Italy from Galliformes birds in 1960s.

Noteworthy, mallards and coots belong to two sympatric species—*Anas platyrhynchos* and *Fulica atra*, respectively—that share large and overlapping distribution areas in Eurasia and, from an ecological point of view, live and interact with each other in the same local communities in wetland habitats, enabling water-mediated transmission of AIVs. The HA gene divergence observed between H10 AIVs from these two sympatric species of waterbirds sharing the same environment appears to confirm that distinct influenza gene pools can be maintained in ducks and coots, as previously hypothesised [17]. This is supported by the nucleotide identity results, showing that the two Italian H10N8 strains isolated from coots were different from those isolated from mallards, also in the nucleotide sequences of the internal genes, except for the MP and NS. It is well documented that distinct gene pools of AIVs can be maintained in gulls, shorebirds, and ducks [127,128,129]. Additionally, previous serological data—obtained between 1992 and 1998 from wild waterbirds sampled in the same protected areas of Central Italy [17]—already indicated some species-specific differences in AIV subtype circulation in these sympatric species. In fact, 407 sera collected from mallards showed HI antibodies against eight of the fourteen HA subtypes tested (except for H3, H4, H7, and H12), while 449 coot sera were only positive for the H3 and H10 subtypes. During the six sampling periods in the Tuscany region (spanning from November/December 1992 to January/March 1998), H10-seropositive coots were found only during the first three, including the H10N8 isolation period [17,130]. 

Multiple mutations in avian influenza genes can alter viral biological characteristics, making them potentially zoonotic. The acquisition of specific mutations in the viral HA can alter the receptor-binding specificity of influenza viruses, thus representing a key factor in interspecies transmission. Various studies have reported amino acid substitutions, including the Q226L and G228S changes (H3 numbering), that cause a change in receptor-binding preference from the avian-type α-2,3 receptor to the human-type α-2,6 receptor [35,131]. Our molecular data showed that the Q226 and G228 residues in the receptor-binding site were highly conserved in all H10 strains, suggesting their preference for binding to avian-type receptors, although the S221P mutation, previously associated with H5N1 increased binding to the human-type α-2,6 receptor [48], was found in all H10 isolates. Further research is needed to better define the effect of this mutation in H10 subtype viruses. Additionally, the HA amino acid sequences at the cleavage site of our H10 strains were typical of low-pathogenicity AIVs. Several mutations of internal genes may play an important role in the mammalian adaptation and, consequently, in the pandemic potential of AIVs [36]. In this regard, full-genome sequence analyses of our H10 strains did not reveal the presence of the two changes in PB2 (E627K and D701N) that are more frequently associated with the adaptation of AIVs to mammalian hosts [132]. Interestingly, the PB2-A588V amino acid substitution, known to be linked to increased virulence in mice infected with H10N8 subtype viruses [47], was found in both our H10N8 strains: A/Eurasian Coot/Italy/125/1994 and A/Eurasian Coot/Italy/114/1995. Additional mutations in other genes, associated with increased pathogenicity in mammals but found in other AIV subtypes [36] were also detected in our H10NX isolates. Overall, mutations/motifs found in the PB1 (2/0), PA (3/0), NP (3/0), and M1 (3/0) were equally distributed among all H10 strains (Table 5). All mutations/motifs found in PB2 (4/2) and NS1 (3/2) were detected in coot strains. In contrast, in seven mallard strains, PB2 mutations/motifs ranged from 2/2 (in four isolates) to 3/2 (in three isolates), whereas NS1 mutations/motifs ranged from 3/2 (in six isolates) to 2/0 (in one isolate). The effects of multiple mutations are very complex to understand [94,133] and could differ among various influenza virus subtypes [105,134]. To our knowledge, the phenotypic effects of these mutations have not been described for the H10 subtype.

Antivirals represent the first line of defence against AIV infections in humans, and their usage is important for reducing pandemic risk [135]. However, treatment and prophylaxis of AIV infections in humans could lead to the emergence of drug-resistant viruses [136]. In our study, the Italian H10NX isolates were shown to be sensitive to oseltamivir and zanamivir when tested by genotypic and phenotypic assays. The NA-D198N amino acid substitution, known to be associated with normal/reduced inhibition (NI/RI) by oseltamivir and zanamivir in human influenza B viruses [137] and detected in the framework region of the NA active site of the H10N7 strain under study, can be attributed to a conserved characteristic of N7 neuraminidase from AIVs [83]. Moreover, no amino acid substitutions at positions 26, 27, 30, 31, and 34 of the M2 gene were found in our H10NX isolates, confirming their susceptibility to adamantanes as well [102]. Finally, all the nine H10 isolates did not possess the most clinically relevant genetic change, I38T, in the PA gene [138], as well as other frequent markers associated with reduced susceptibility to baloxavir marboxil in seasonal human influenza strains [103].

The main limitation of our study is the restricted number of sequences from coots that were available for analysis. Further sample collections from this waterbird species are needed to better define the apparent gene pool divergences between coots and ducks.

## 5. Conclusions

This retrospective study, based on molecular characterisation of nine H10 AIV isolates obtained in Italy from wild mallards (1 H10N2 and 5 H10N7), domestic mallards (1 H10N7), and Eurasian coots (2 H10N8), demonstrated that, despite sharing the same environment, sympatric dabbling ducks and coots could harbour different H10 gene pools. This hypothesis can be supported either by the high diversity existing between the two sampled species [139] or by their use of different trophic niches, where mallards predominantly feed at the water surface whereas Eurasian coots also feed in deep-water habitats [125,126]. Notably, our coot isolates grouped with an H10N8 coot strain isolated in 2010 in Germany, that—according to Italian recapture data of coots ringed abroad—represents a possible reproductive site of coots wintering in Italy [126]. Moreover, the HA genes of three H10N7 viruses obtained from wild mallards were closely related to that of an H10N7 AIV isolated from domestic mallards, thus indicating a possible bidirectional transmission of AIVs between outdoor-housed ducks and wild waterfowl. Finally, although several potential zoonotic molecular markers were observed in our strains, the only PB2-A588V substitution, experimentally found to promote the mammalian adaptation of H10, was found in both H10N8 coot strains.

## Figures and Tables

**Table 1:** H10Nx viruses under study, isolated in Italy from wild and domestic waterbirds (1994–2007).

AIV Isolate	HN Subtype	Sampling Date dd/mm/yy	Sampling Site	Bird			
				Species	Sex	Age	Origin
A/Eurasian Coot/It/125/1994	H10N8	11 January 1994	Orbetello Lagoon ^	*Fulica atra*	M	Ad	W
A/Eurasian Coot/It/114/1995	H10N8	12 December 1995	Burano Lake ^	*F. atra*	F	Ad	W
A/Mallard/It/90/2002	H10N2	30 October 2002	Orbetello Lagoon	*Anas platyrhynchos*	M	Juv	W
A/Mallard/It/166998/2005 ^§^	H10N7	21 July 2005	Bagnacavallo *	*A. platyrhynchos*	Na	Na	D
A/Mallard/It/Eco-634/2005	H10N7	28 December 2005	Orbetello Lagoon	*A. platyrhynchos*	F	Juv	W
A/Mallard/It/Eco-7/2006	H10N7	23 January 2006	Orbetello Lagoon	*A. platyrhynchos*	F	Juv	W
A/Mallard/It/Eco-33/2006	H10N7	24 January 2006	Orbetello Lagoon	*A. platyrhynchos*	M	Juv	W
A/Mallard/It/Eco-360/2006	H10N7	24 November 2006	Orbetello Lagoon	*A. platyrhynchos*	M	Juv	W
A/Mallard/It/195376/2007	H10N7	31 July 2007	Casola Valsenio *	*A. platyrhynchos*	Na	Na	W

^ Grosseto province, Tuscany region, Central Italy; * Ravenna province, Emilia-Romagna region, Northern Italy; It, Italy; ^§^, AIV isolate obtained from ten pooled samples; M, male; F, female; Juv, juvenile; Ad, adult; W, wild; D, domestic; Na, not available.

**Table 2:** BLAST (GISAID) results for HA sequences from H10NX strains under study (updated 24 June 2025).

H10NX Strains	BLAST Results	Nt Identity
A/Eurasian Coot/It/125/1994 (H10N8)	A/Eurasian coot/Germany/R411/2010 (H10N8)	96%
A/Eurasian Coot/It/114/1995 (H10N8)	A/Eurasian coot/Germany/R411/2010 (H10N8)	96%
A/Mallard/It/90/2002 (H10N2)	A/mallard/Sweden/1417/2002 (H10N7)	98%
A/Mallard/It/166998/2005 (H10N7)	A/shoveler/Egypt/09781-NAMRU3/2004 (H10N7)	99%
A/Mallard/It/Eco-634/2005 (H10N7)	A/shoveler/Egypt/09781-NAMRU3/2004 (H10N7)	98%
A/Mallard/It/Eco-7/2006 (H10N7)	A/shoveler/Egypt/09781-NAMRU3/2004 (H10N7)	98%
A/Mallard/It/Eco-33/2006 (H10N7)	A/shoveler/Egypt/09781-NAMRU3/2004 (H10N7)	98%
A/Mallard/It/Eco-360/2006 (H10N7)	A/Mallard/Italy/46341-12/2006 (H10N7)	99%
A/Mallard/It/195376/2007 (H10N7)	A/Mallard/Germany/R2075/2007 (H10N7)	99%

Nt, nucleotide; It, Italy.

**Table 3:** Hemagglutinin cleavage site of H10 influenza viruses isolated from coots and mallards.

Viruses	Subtype	Hemagglutinin Cleavage Site	
		Amino Acids	Nucleotides
A/Eurasian Coot/It/125/1994	H10N8	PE**VV**QGR/GLF	CCAGAAGTAGTGCAAGGAAGGGGTTTGTTT
A/Eurasian Coot/It/114/1995	H10N8	PE**VV**QGR/GLF	CCAGAAGTAGTGCAAGGAAGGGGTTTGTTT
A/Mallard/It/90/2002	H10N2	PE**IM**QGR/GLF	CCAGAAATAATGCAAGGGAGAGGTCTATTT
A/Mallard/It/166998/2005	H10N7	PE**IM**QGR/GLF	CCAGAAATAATGCAAGGGAGAGGTCTATTT
A/Mallard/It/Eco-634/2005	H10N7	PE**IM**QGR/GLF	CCAGAAATAATGCAAGGGAGAGGTCTATTT
A/Mallard/It/Eco-7/2006	H10N7	PE**IM**QGR/GLF	CCAGAAATAATGCAAGGGAGAGGTCTATTT
A/Mallard/It/Eco-33/2006	H10N7	PE**IM**QGR/GLF	CCAGAAATAATGCAAGGGAGAGGTCTATTT
A/Mallard/It/Eco-360/2006	H10N7	PE**IM**QGR/GLF	CCAGAAATAATGCAAGGGAGAGGTCTATTT
A/Mallard/It/195376/2007	H10N7	PE**IM**QGR/GLF	CCAGAAATAATGCAAGGGAGAGGTCTATTT

In bold, amino acid differences; It, Italy.

**Table 4:** Receptor-binding site of H10 avian influenza isolates.

Viruses	Receptor-Binding Site (Amino Acidic Positions—H10 Numbering)								
	105	161	163	193	200	204	205	Left Edge 230–239	Right Edge 142–146
A/Eurasian Coot/It/125/1994	Y	W	V	H	E	L	Y	RPQVNGQSGR	G**V**TKA
A/Eurasian Coot/It/114/1995	Y	W	V	H	E	L	Y	RPQVNGQSGR	G**V**TKA
A/Mallard/It/90/2002	Y	W	V	H	E	L	Y	RPQVNGQSGR	GTTKA
A/Mallard/It/166998/2005	Y	W	V	H	E	L	Y	RPQVNGQSGR	GTTKA
A/Mallard/It/Eco-634/2005	Y	W	V	H	E	L	Y	RPQVNGQSGR	GTTKA
A/Mallard/It/Eco-7/2006	Y	W	V	H	E	L	Y	RPQVNGQSGR	GTTKA
A/Mallard/It/Eco-33/2006	Y	W	V	H	E	L	Y	RPQVNGQSGR	GTTKA
A/Mallard/It/Eco-360/2006	Y	W	V	H	E	L	Y	RPQVNGQSGR	GTTKA
A/Mallard/It/195376/2007	Y	W	V	H	E	L	Y	RPQVNGQSGR	GTTKA

In bold, valine amino acid detected only in Eurasian coots; It, Italy.

**Table 5:** Distribution of potentially zoonotic markers in internal genes of H10 strains isolated in Italy from 1994 to 2007. See Table 1, Table A1 and Table S4 for details.

**Genes**	**No. of** **Mutation/Motif**	**Mutation/Motif Grouped by Bird Species, and Sampling dd/mm/yy of Nine H10 Isolates**								
		**Eurasian Coot**		**Mallard**						
		11 January 1994 ^ **H10N8 ^(1)^**	12 December 1995 **H10N8 ^(2)^**	30 October 2002 **H10N2 ^(3)^**	21 July 2005 H10N7 *^,(4)^	28 December 2005 **H10N7 ^(5)^**	23 January 2006 **H10N7 ^(6)^**	24 January 2006 **H10N7 ^(7)^**	24 November 2006 **H10N7 ^(8)^**	31 July 2007 **H10N7 ^(9)^**
PB2	4/2	4/2	4/2	2/2	2/2	3/2	3/2	3/2	2/2	2/2
PB1	2/0	2/0	2/0	2/0	2/0	2/0	2/0	2/0	2/0	2/0
PA	3/0	3/0	3/0	3/0	3/0	3/0	3/0	3/0	3/0	3/0
NP	3/0	1/0	1/0	1/0	2/0	2/0	2/0	3/0	2/0	2/0
M1	3/0	3/0	3/0	3/0	3/0	3/0	3/0	3/0	3/0	3/0
NS1	3/2	3/2	3/2	3/2	3/2	3/2	3/2	3/2	3/2	2/0

^, dd/mm/yy; ^(1)^, A/Eurasian Coot/It/125/1994; ^(2)^, A/Eurasian Coot/It/114/1995; ^(3)^, A/Mallard/It/90/2002; ^(4)^, A/Mallard/It/166998/2005; ^(5)^, A/Mallard/It/Eco-634/2005; ^(6)^, A/Mallard/It/Eco-7/2006; ^(7)^, A/Mallard/It/Eco-33/2006; ^(8)^, A/Mallard/It/Eco-360/2006; ^(9)^, A/Mallard/It/195376/2007; *, pooled samples collected from domestic mallards.

**Table 6:** H10NX avian influenza virus susceptibility to oseltamivir and zanamivir tested by phenotypic assay.

Isolate	Subtype	Mean IC_50_ ± SD (nM) *	
		Oseltamivir	Zanamivir
A/Eurasian Coot/It/125/1994	H10N8	3.4 ± 0.07	1.3 ± 0.02
A/Eurasian Coot/It/114/1995	H10N8	3.2 ± 0.07	1.1 ± 0.01
A/Mallard/It/90/2002	H10N2	0.3 ± 0.001	0.7 ± 0.04
A/Mallard/It/Eco-634/2005	H10N7	0.7 ± 0.03	1 ± 0.06
A/Mallard/It/Eco-7/2006	H10N7	0.8 ± 0.07	1 ± 0.002
A/Mallard/It/Eco-33/2006	H10N7	1.1 ± 0.07	1.3 ± 0.02
A/Mallard/It/Eco-360/2006	H10N7	1.2 ± 0.07	1.2 ± 0.06
A/Mallard/It/195376/2007	H10N7	0.7 ± 0.09	0.9 ± 0.02
A/Victoria/4897/2022 (Wt)	H1N1pdm09	1.1 ± 0.01	0.3 ± 0.006
A/Darwin/9/2021 (Wt)	H3N2	0.3 ± 0.06	0.3 ± 0.002
H1-H275Y ^	H1N1pdm09	492.9 ± 0.07	0.7 ± 0.04

*, concentration that inhibits viral NA activity by 50% expressed as the mean ± standard deviation (SD); It, Italy; Wt, wild-type reference virus; ^, oseltamivir-resistant virus with NA-H275Y mutation.

## Data Availability

Sequence accession numbers in GISAID (Global Initiative on Sharing Avian Flu Data) are as follows: EPI_ISL_20096817 for A/Eurasian Coot/Italy/125/1994; EPI_ISL_20096818 for A/Eurasian Coot/Italy/114/1995; EPI_ISL_20096819 for A/Mallard/Italy/90/2002; EPI_ISL_20096820 for A/Mallard/Italy/166998/2005; EPI_ISL_20096822 for A/Mallard/Italy/Eco-634/2005; EPI_ISL_20096945 for A/Mallard/Italy/Eco-7/2006; EPI_ISL_20097047 for A/Mallard/Italy/Eco-33/2006; EPI_ISL_20097048 for A/Mallard/Italy/Eco-360/2006; and EPI_ISL_20097050 for A/Mallard/Italy/195376/2007.

## Supplementary Materials

The following supporting information can be downloaded at: https://www.mdpi.com/article/10.3390/microorganisms13112575/s1. Figure S1. HA genes similarity in avian H10NX strains under study. Figure S2. NA genes similarity in avian H10NX strains under study. Figure S3. NP genes similarity in avian H10NX strains under study. Figure S4. PA genes similarity in avian H10NX strains under study. Figure S5. PB1 genes similarity in avian H10NX strains under study. Figure S6. PB2 genes similarity in avian H10NX strains under study. Figure S7. MP genes similarity in avian H10NX strains under study. Figure S8. NS genes similarity in avian H10NX strains under study. Figure S9. Names of H10NX strains used in the HA phylogenetic tree shown in Appendix A (Figure A1). The phylogenetic tree was generated using the maximum likelihood algorithm with 5000 bootstrap replicates. Table S1. Summary of HA mutations screened in the nine H10 AIV strains under study (Italy 1994–2007) and known to be associated with zoonotic potential, as previously reviewed [36]. Table S2. Summary of N2, N7, and N8 mutations screened in the nine H10 AIV strains under study (Italy 1994–2007) and known to be associated with reduced susceptibility to oseltamivir and zanamivir drugs, as previously reviewed [36,83]. Table S3. Summary of NA mutations screened in the nine H10 AIV strains under study (Italy 1994–2007) and known to be associated with zoonotic potential, as previously reported [36]. Table S4. Multiple mutations detected in internal protein genes of the nine H10NX AIVs under study. In light blue cells, the amino acid substitutions previously reported to be linked with increased replication in mammalian and avian cells, and with increased virulence in mice, ducks, chickens, and pigs. Table S5. GISAID accession numbers of HA sequences used in the study.

## Appendix A

**Table A1:** Multiple mutations detected in internal protein genes of the nine H10NX AIVs under study and phenotypic effects previously reported in the literature.

Protein	Mutation/ Motif	Strains with Mutation	Phenotypic Effect/Subtype Tested	Reference
PB2	R340K	A/Eurasian Coot/It/125/1994, A/Eurasian Coot/It/114/1995, A/Mallard/It/Eco-634/2005, A/Mallard/It/Eco-7/2006, and A/Mallard/It/Eco-33/2006	Increased virulence in mice	[105]
	K389R	All	Increased polymerase activity and replication in mammalian cell line/H7N9	[106]
	A588V	A/Eurasian Coot/It/125/1994 and A/Eurasian Coot/It/114/1995	Increased polymerase activity and replication in avian and mammalian cell line; increased virulence in mice/H7N9, H9N2, and H10N8	[105]
	V598T	All	Increased polymerase activity and replication in mammalian cell line; increased virulence in mice/H7N9	[106]
	L89V, G309D	All	Increased polymerase activity and replication in mammalian cell line; increased virulence in mice/H5N1	[107]
	L89V, G309D, T339K, R477G, I495V, K627E, and A676T	All	Increased polymerase activity and replication in mammalian cell line; increased virulence in mice/H5N1	[107]
PB1	D3V	All	Increased polymerase activity and replication in avian and mammalian cell line/H5N1	[108]
	D622G	All	Increased polymerase activity and virulence in mice/H5N1	[109]
PA	S37A	All	Increased polymerase activity in mammalian cell line/H7N9	[110]
	N383D	All	Increased polymerase activity in avian and mammalian cell line/H5N1	[111]
	N409S	All	Increased polymerase activity in avian and mammalian cell line/H7N9	[110]
NP	M105V	A/Mallard/It/166998/2005, A/Mallard/It/Eco-634/2005, A/Mallard/It/Eco-7/2006, A/Mallard/It/Eco-33/2006, A/Mallard/It/Eco-360/2006, and A/Mallard/It/195376/2007	Increased virulence in chickens/H5N1	[112]
	I109T	A/Mallard/It/Eco-33/2006	Increased polymerase activity and viral replication in chickens (but not in ducks), and increased virulence in chickens/H5N1	[112]
	A184K	All	Increased replication in avian cells virulence in chickens and enhanced IFN response/H5N1	[113]
M1	N30D	All	Increased virulence in mice/H5N1	[114]
	I43M	All	Increased virulence in mice, ducks, and chickens/H5N1	[115]
	T215A	All	Increased virulence in mice/H5N1	[114]
NS1	P42S	A/Eurasian Coot/It/125/1994, A/Eurasian Coot/It/114/1995, A/Mallard/It/90/2002, A/Mallard/It/166998/2005, A/Mallard/It/Eco-634/2005, A/Mallard/It/Eco-7/2006, A/Mallard/It/Eco-33/2006, and A/Mallard/It/Eco-360/2006	Increased virulence in mice and pigs/H5N1	[116]
	C138F	All	Increased replication in mammalian cells and decreased interferon response/H5N1	[117]
	V149A	All	Increased virulence and decreased interferon response in chickens/H5N1	[117]
	L103F, I106M	A/Eurasian Coot/It/125/1994, A/Eurasian Coot/It/114/1995, A/Mallard/It/90/2002, A/Mallard/It/166998/2005, A/Mallard/It/Eco-634/2005, A/Mallard/It/Eco-7/2006, A/Mallard/It/Eco-33/2006, and A/Mallard/It/Eco-360/2006	Increased virulence in mice	[118]
	K55E, K66E, C138F	A/Eurasian Coot/It/125/1994, A/Eurasian Coot/It/114/1995, A/Mallard/It/90/2002, A/Mallard/It/166998/2005, A/Mallard/It/Eco-634/2005, A/Mallard/It/Eco-7/2006, A/Mallard/It/Eco-33/2006, and A/Mallard/It/Eco-360/2006	Increased replication in mammalian cells and decreased IF response/H5N1	[117,119]

## References

[1] Webster R.G., Bean W.J., Gorman O.T., Chambers T.M., Kawaoka Y. (1992). Evolution and ecology of influenza A viruses. Microbiol. Rev..

[2] Webster R.G., Govorkova E.A. (2014). Continuing challenges in influenza. Ann. N. Y. Acad. Sci..

[3] Donatelli I., Castrucci M.R., De Marco M.A., Delogu M., Webster R.G. (2017). Human-Animal Interface: The Case for Influenza Interspecies Transmission. Adv. Exp. Med. Biol..

[4] Matrosovich M.N., Gambaryan A.S., Teneberg S., Piskarev V.E., Yamnikova S.S., Lvov D.K., Robertson J.S., Karlsson K.A. (1997). Avian influenza A viruses differ from human viruses by recognition of sialyloligosaccharides and gangliosides and by a higher conservation of the HA receptor-binding site. Virology.

[5] Runstadler J.A., Puryear W.B. (2024). The virus is out of the barn: The emergence of HPAI as a pathogen of avian and mammalian wildlife around the globe. Am. J. Vet. Res..

[6] World Organisation for Animal Health (WOAH) Terrestrial Animal Health Code, SECTION: 10. AVES, Chapter: 10.4. Infection with High Pathogenicity Avian Influenza Viruses. https://sont.woah.org/portal/tool?le=en.

[7] Abdelwhab E.M., Mettenleiter T.C. (2023). Zoonotic Animal Influenza Virus and Potential Mixing Vessel Hosts. Viruses.

[8] Everest H., Billington E., Daines R., Burman A., Iqbal M. (2021). The Emergence and Zoonotic Transmission of H10Nx Avian Influenza Virus Infections. mBio.

[9] Berg M., Englund L., Abusugra I.A., Klingeborn B., Linné T. (1990). Close relationship between mink influenza (H10N4) and concomitantly circulating avian influenza viruses. Arch. Virol..

[10] Si Y.J., Park Y.R., Baek Y.G., Park M.J., Lee E.K., Lee K.N., Kim H.R., Lee Y.J., Lee Y.N. (2022). Pathogenesis and genetic characteristics of low pathogenic avian influenza H10 viruses isolated from migratory birds in South Korea during 2010–2019. Transbound. Emerg. Dis..

[11] Pan American Health Organization (2004). Avian Influenza Virus A (H10N7) Circulating Among Humans in Egypt. https://www.paho.org/en/documents/avian-influenza-virus-h10n7-circulating-among-humans-egypt-vol-2-no-18-7-may-2004.

[12] Arzey G.G., Kirkland P.D., Arzey K.E., Frost M., Maywood P., Conaty S., Hurt A.C., Deng Y.M., Iannello P., Barr I. (2012). Influenza virus A (H10N7) in chickens and poultry abattoir workers, Australia. Emerg. Infect. Dis..

[13] Chen H., Yuan H., Gao R., Zhang J., Wang D., Xiong Y., Fan G., Yang F., Li X., Zhou J. (2014). Clinical and epidemiological characteristics of a fatal case of avian influenza A H10N8 virus infection: A descriptive study. Lancet.

[14] Xu Y., Cao H., Liu H., Sun H., Martin B., Zhao Y., Wang Q., Deng G., Xue J., Zong Y. (2015). Identification of the source of A (H10N8) virus causing human infection. Infect. Genet. Evol..

[15] Fusaro A., Gonzales J.L., Kuiken T., Mirinavičiūtė G., Niqueux É., Ståhl K., Staubach C., European Food Safety Authority, European Centre for Disease Prevention and Control, European Union Reference Laboratory for Avian Influenza (2024). Avian influenza overview December 2023–March 2024. EFSA J..

[16] ECDC Weekly Bulletin, Communicable Disease Threats Report, 10–16 May 2025, Week 20. https://www.ecdc.europa.eu/en/publications-data/communicable-disease-threats-report-10-16-may-2025-week-20.

[17] De Marco M.A., Campitelli L., Foni E., Raffini E., Barigazzi G., Delogu M., Guberti V., Di Trani L., Tollis M., Donatelli I. (2004). Influenza surveillance in birds in Italian wetlands (1992–1998): Is there a host restricted circulation of influenza viruses in sympatric ducks and coots?. Vet. Microbiol..

[18] Delogu M., De Marco M.A., Di Trani L., Raffini E., Cotti C., Puzelli S., Ostanello F., Webster R.G., Cassone A., Donatelli I. (2010). Can preening contribute to influenza A virus infection in wild waterbirds?. PLoS ONE.

[19] De Marco M.A., Delogu M., Facchini M., Di Trani L., Boni A., Cotti C., Graziosi G., Venturini D., Regazzi D., Ravaioli V. (2021). Serologic Evidence of Occupational Exposure to Avian Influenza Viruses at the Wildfowl/Poultry/Human Interface. Microorganisms.

[20] Council of European Union (1992). Council Directive 92/40/EEC of 19 May 1992 Introducing Community Measures for the Control of Avian Influenza. Off. J. L.

[21] World Health Organization (2002). WHO Manual on Animal Influenza Diagnosis and Surveillance. https://apps.who.int/iris/bitstream/handle/10665/68026/WHO_CDS_CSR_NCS_2002.5.pdf.

[22] Siebinga J.T., de Boer G.F. (1988). Influenza A viral nucleoprotein detection in isolates from human and various animal species. Arch. Virol..

[23] Fouchier R.A., Bestebroer T.M., Herfst S., Van Der Kemp L., Rimmelzwaan G.F., Osterhaus A.D. (2000). Detection of influenza A viruses from different species by PCR amplification of conserved sequences in the matrix gene. J. Clin. Microbiol..

[24] Zhou B., Donnelly M.E., Scholes D.T., St George K., Hatta M., Kawaoka Y., Wentworth D.E. (2009). Single-reaction genomic amplification accelerates sequencing and vaccine production for classical and Swine origin human influenza a viruses. J. Virol..

[25] Bolger A.M., Lohse M., Usadel B. (2014). Trimmomatic: A flexible trimmer for Illumina sequence data. Bioinformatics.

[26] Shepard S.S., Meno S., Bahl J., Wilson M.M., Barnes J., Neuhaus E. (2016). Viral deep sequencing needs an adaptive approach: IRMA, the iterative refinement meta-assembler. BMC Genom..

[27] Katoh K., Misawa K., Kuma K., Miyata T. (2002). MAFFT: A novel method for rapid multiple sequence alignment based on fast Fourier transform. Nucleic Acids Res..

[28] Thompson J.D., Gibson T.J., Plewniak F., Jeanmougin F., Higgins D.G. (1997). The CLUSTAL_X windows interface: Flexible strategies for multiple sequence alignment aided by quality analysis tools. Nucleic Acids Res..

[29] Hall T.A. (1999). BioEdit: A user-friendly biological sequence alignment editor and analysis pro-gram for Windows 95/98/NT. Nucleic Acids Symp. Ser..

[30] Nguyen L.T., Schmidt H.A., von Haeseler A., Minh B.Q. (2015). IQ-TREE: A fast and effective stochastic algorithm for estimating maximum-likelihood phylogenies. Mol. Biol. Evol..

[31] Puzelli S., Facchini M., Di Martino A., Fabiani C., Lackenby A., Zambon M., Donatelli I. (2011). Evaluation of the antiviral drug susceptibility of influenza viruses in Italy from 2004/05 to 2009/10 epidemics and from the recent 2009 pandemic. Antivir. Res..

[32] World Health Organization (2012). Meetings of the WHO working group on surveillance of influenza antiviral susceptibility—Geneva, November 2011 and June 2012. Wkly. Epidemiol. Rec..

[33] Berhane Y., Joseph T., Lung O., Embury-Hyatt C., Xu W., Cottrell P., Raverty S. (2022). Isolation and Characterization of Novel Reassortant Influenza A(H10N7) Virus in a Harbor Seal, British Columbia, Canada. Emerg. Infect. Dis..

[34] Lv X., Tian J., Li X., Bai X., Li Y., Li M., An Q., Song X., Xu Y., Sun H. (2023). H10Nx avian influenza viruses detected in wild birds in China pose potential threat to mammals. One Health.

[35] Hao M., Wu J., Ji L., Zhao Y., Zhang S., Guan Y., Li L., Yang W., Zhang Y., Chen J. (2025). Pathogenicity, transmissibility, and receptor binding of a human-isolated influenza A (H10N5) virus. mBio.

[36] Suttie A., Deng Y.M., Greenhill A.R., Dussart P., Horwood P.F., Karlsson E.A. (2019). Inventory of molecular markers affecting biological characteristics of avian influenza A viruses. Virus Genes.

[37] Sun X., Belser J.A., Yang H., Pulit-Penaloza J.A., Pappas C., Brock N., Zeng H., Creager H.M., Stevens J., Maines T.R. (2019). Identification of key hemagglutinin residues responsible for cleavage, acid stability, and virulence of fifth-wave highly pathogenic avian influenza A(H7N9) viruses. Virology.

[38] Su Y., Yang H.-Y., Zhang B.-J., Jia H.-L., Tien P. (2008). Analysis of a point mutation in H5N1 avian influenza virus hemagglutinin in relation to virus entry into live mammalian cells. Arch. Virol..

[39] Wang W., Lu B., Zhou H., Suguitan A.L., Cheng X., Subbarao K., Kemble G., Jin H. (2010). Glycosylation at 158N of the hemagglutinin protein and receptor binding specificity synergistically affect the antigenicity and immunogenicity of a live attenuated H5N1 A/Vietnam/1203/2004 vaccine virus in ferrets. J. Virol..

[40] Yang Z.Y., Wei C.J., Kong W.P., Wu L., Xu L., Smith D.F., Nabel G.J. (2007). Immunization by avian H5 influenza hemagglutinin mutants with altered receptor binding specificity. Science.

[41] Naughtin M., Dyason J.C., Mardy S., Sorn S., von Itzstein M., Buchy P. (2011). Neuraminidase inhibitor sensitivity and receptor-binding specificity of Cambodian clade 1 highly pathogenic H5N1 influenza virus. Antimicrob. Agents Chemother..

[42] Kongchanagul A., Suptawiwat O., Kanrai P., Uiprasertkul M., Puthavathana P., Auewarakul P. (2008). Positive selection at the receptor-binding site of haemagglutinin H5 in viral sequences derived from human tissues. J. Gen. Virol..

[43] Yamada S., Suzuki Y., Suzuki T., Le M.Q., Nidom C.A., Sakai-Tagawa Y., Muramoto Y., Ito M., Kiso M., Horimoto T. (2006). Haemagglutinin mutations responsible for the binding of H5N1 influenza A viruses to human-type receptors. Nature.

[44] Gao Y., Zhang Y., Shinya K., Deng G., Jiang Y., Li Z., Guan Y., Tian G., Li Y., Shi J. (2009). Identification of amino acids in HA and PB2 critical for the transmission of H5N1 avian influenza viruses in a mammalian host. PLoS Pathog..

[45] Chen L.M., Blixt O., Stevens J., Lipatov A.S., Davis C.T., Collins B.E., Cox N.J., Paulson J.C., Donis R.O. (2012). In vitro evolution of H5N1 avian influenza virus toward human-type receptor specificity. Virology.

[46] Han P., Hu Y., Sun W., Zhang S., Li Y., Wu X., Yang Y., Zhu Q., Jiang T., Li J. (2015). Mouse lung-adapted mutation of E190G in hemagglutinin from H5N1 influenza virus contributes to attenuation in mice. J. Med. Virol..

[47] Watanabe Y., Ibrahim M.S., Ellakany H.F., Kawashita N., Mizuike R., Hiramatsu H., Sriwilaijaroen N., Takagi T., Suzuki Y., Ikuta K. (2011). Acquisition of human-type receptor binding specificity by new H5N1 influenza virus sublineages during their emergence in birds in Egypt. PLoS Pathog..

[48] Behera A.K., Chandra I., Cherian S.S. (2016). Molecular dynamics simulation of the effects of single (S221P) and double (S221P and K216E) mutations in the hemagglutinin protein of influenza A H5N1 virus: A study on host receptor specificity. J. Biomol. Struct. Dyn..

[49] Chutinimitkul S., van Riel D., Munster V.J., van den Brand J.M., Rimmelzwaan G.F., Kuiken T., Osterhaus A.D., Fouchier R.A., de Wit E. (2010). In vitro assessment of attachment pattern and replication efficiency of H5N1 influenza A viruses with altered receptor specificity. J. Virol..

[50] Stevens J., Blixt O., Tumpey T.M., Taubenberger J.K., Paulson J.C., Wilson I.A. (2006). Structure and receptor specificity of the hemagglutinin from an H5N1 influenza virus. Science.

[51] Herfst S., Schrauwen E.J., Linster M., Chutinimitkul S., de Wit E., Munster V.J., Sorrell E.M., Bestebroer T.M., Burke D.F., Smith D.J. (2012). Airborne transmission of influenza A/H5N1 virus between ferrets. Science.

[52] Linster M., van Boheemen S., de Graaf M., Schrauwen E.J.A., Lexmond P., Mänz B., Bestebroer T.M., Baumann J., van Riel D., Rimmelzwaan G.F. (2014). Identification, characterization, and natural selection of mutations driving airborne transmission of A/H5N1 virus. Cell.

[53] Wessels U., Abdelwhab E.M., Veits J., Hoffmann D., Mamerow S., Stech O., Stech J. (2018). A dual motif in the hemagglutinin of H5N1 goose/Guangdong-like highly pathogenic avian influenza virus strains is conserved from their early evolution and increases both membrane fusion pH and virulence. J. Virol..

[54] Abdelwhab E.-S.M., Veits J., Breithaupt A., Gohrbandt S., Ziller M., Teifke J.P., Stech J., Mettenleiter T.C. (2016). Prevalence of the C-terminal truncations of NS1 in avian influenza A viruses and effect on virulence and replication of a highly pathogenic H7N1 virus in chickens. Virulence.

[55] Reed M.L., Yen H.L., DuBois R.M., Bridges O.A., Salomon R., Webster R.G., Russell C.J. (2009). Amino acid residues in the fusion peptide pocket regulate the pH of activation of the H5N1 influenza virus hemagglutinin protein. J. Virol..

[56] Krenn B.M., Egorov A., Romanovskaya-Romanko E., Wolschek M., Nakowitsch S., Ruthsatz T., Kiefmann B., Morokutti A., Humer J., Geiler J. (2011). HA2 mutation increases the infectivity and immunogenicity of a live attenuated H5N1 intranasal influenza vaccine candidate lacking NS1. PLoS ONE.

[57] Ilyushina N.A., Govorkova E.A., Gray T.E., Bovin N.V., Webster R.G. (2008). Human-like receptor specificity does not affect the neuraminidase-inhibitor susceptibility of H5N1 influenza viruses. PLoS Pathog..

[58] Yen H.L., Aldridge J.R., Boon A.C., Ilyushina N.A., Salomon R., Hulse-Post D.J., Marjuki H., Franks J., Boltz D.A., Bush D. (2009). Changes in H5N1 influenza virus hemagglutinin receptor binding domain affect systemic spread. Proc. Natl. Acad. Sci. USA.

[59] Peng W., Bouwman K.M., McBride R., Grant O.C., Woods R.J., Verheije M.H., Paulson J.C., de Vries R.P. (2018). Enhanced human-type receptor binding by ferret-transmissible H5N1 with a K193T mutation. J. Virol..

[60] Maines T.R., Chen L.M., Van Hoeven N., Tumpey T.M., Blixt O., Belser J.A., Gustin K.M., Pearce M.B., Pappas C., Stevens J. (2011). Effect of receptor binding domain mutations on receptor binding and transmissibility of avian influenza H5N1 viruses. Virology.

[61] Guo H., de Vries E., McBride R., Dekkers J., Peng W., Bouwman K.M., Nycholat C., Verheije M.H., Paulson J.C., van Kuppeveld F.J. (2017). Highly pathogenic influenza A(H5Nx) viruses with altered H5 receptor-binding specificity. Emerg. Infect. Dis..

[62] Webster R.G., Rott R. (1987). Influenza virus A pathogenicity: The pivotal role of hemagglutinin. Cell.

[63] Imai M., Watanabe T., Hatta M., Das S.C., Ozawa M., Shinya K., Zhong G., Hanson A., Katsura H., Watanabe S. (2012). Experimental adaptation of an influenza H5 HA confers respiratory droplet transmission to a reassortant H5 HA/H1N1 virus in ferrets. Nature.

[64] Dortmans J.C., Dekkers J., Wickramasinghe I.N., Verheije M.H., Rottier P.J., van Kuppeveld F.J., de Vries E., de Haan C.A. (2013). Adaptation of novel H7N9 influenza A virus to human receptors. Sci. Rep..

[65] Xu R., de Vries R.P., Zhu X., Nycholat C.M., McBride R., Yu W., Paulson J.C., Wilson I.A. (2013). Preferential recognition of avian-like receptors in human influenza A H7N9 viruses. Science.

[66] Ramos I., Krammer F., Hai R., Aguilera D., Bernal-Rubio D., Steel J., García-Sastre A., Fernandez-Sesma A. (2013). H7N9 influenza viruses interact preferentially with α2,3-linked sialic acids and bind weakly to α2,6-linked sialic acids. J. Gen. Virol..

[67] de Vries R.P., Peng W., Grant O.C., Thompson A.J., Zhu X., Bouwman K.M., Paulson J.C. (2017). Three mutations switch H7N9 influenza to human-type receptor specificity. PLoS Pathog..

[68] Srinivasan K., Raman R., Jayaraman A., Viswanathan K., Sasisekharan R. (2013). Quantitative description of glycan-receptor binding of influenza A virus H7 hemagglutinin. PLoS ONE.

[69] Jin F., Dong X., Wan Z., Ren D., Liu M., Geng T., Zhang J., Gao W., Shao H., Qin A. (2019). A single mutation N166D in hemagglutinin affects antigenicity and pathogenesis of H9N2 avian influenza virus. Viruses.

[70] Teng Q., Xu D., Shen W., Liu Q., Rong G., Li X., Yan L., Yang J., Chen H., Yu H. (2016). A single mutation at position 190 in hemagglutinin enhances binding affinity for human-type sialic acid receptor and replication of H9N2 avian influenza virus in mice. J. Virol..

[71] Wan H., Perez D.R. (2007). Amino acid 226 in the hemagglutinin of H9N2 influenza viruses determines cell tropism and replication in human airway epithelial cells. J. Virol..

[72] Ramirez-Nieto G., Monne I., Stevens J., Cattoli G., Capua I., Chen L.M., Donis R.O., Busch J., Paulson J.C., Brockwell C. (2008). Replication and transmission of H9N2 influenza viruses in ferrets: Evaluation of pandemic potential. PLoS ONE.

[73] Sorrell E.M., Wan H., Araya Y., Song H., Perez D.R. (2009). Minimal molecular constraints for respiratory droplet transmission of an avian-human H9N2 influenza A virus. Proc. Natl. Acad. Sci. USA.

[74] Wang F., Qi J., Bi Y., Zhang W., Wang M., Zhang B., Wang M., Liu J., Yan J., Shi Y. (2015). Adaptation of avian influenza A (H6N1) virus from avian to human receptor-binding preference. EMBO J..

[75] de Vries R.P., Tzarum N., Peng W., Thompson A.J., Ambepitiya Wickramasinghe I.N., de la Pena A.T.T., van Breemen M.J., Bouwman K.M., Zhu X., McBride R. (2017). A single mutation in Taiwanese H6N1 influenza hemagglutinin switches binding to human-type receptors. EMBO Mol. Med..

[76] Qu Z., Ma S., Kong H., Deng G., Shi J., Liu L., Chen H. (2017). Identification of a key amino acid in hemagglutinin that increases human-type receptor binding and transmission of an H6N2 avian influenza virus. Microbes Infect..

[77] Song H., Qi J., Xiao H., Bi Y., Zhang W., Xu Y., Wang F., Shi Y., Gao G.F. (2017). Avian-to-Human Receptor-Binding Adaptation by Influenza A Virus Hemagglutinin H4. Cell Rep..

[78] Yu Z., Ren Z., Zhao Y., Cheng K., Sun W., Zhang X., Wu J., He H., Xia X., Gao Y. (2019). PB2 and hemagglutinin mutations confer a virulent phenotype on an H1N2 avian influenza virus in mice. Arch. Virol..

[79] Tzarum N., de Vries R.P., Peng W., Thompson A.J., Bouwman K.M., McBride R., Yu W., Zhu X., Verheije M.H., Paulson J.C. (2017). The 150-Loop Restricts the Host Specificity of Human H10N8 Influenza Virus. Cell Rep..

[80] Zhang H., de Vries R.P., Tzarum N., Zhu X., Yu W., McBride R., Paulson J.C., Wilson I.A. (2015). A human-infecting H10N8 influenza virus retains a strong preference for avian-type receptors. Cell Host Microbe.

[81] Lu X., Qi J., Shi Y., Wang M., Smith D.F., Heimburg-Molinaro J., Zhang Y., Paulson J.C., Xiao H., Gao G.F. (2013). Structure and receptor binding specificity of hemagglutinin H13 from avian influenza A virus H13N6. J. Virol..

[82] Colman P.M., Hoyne P.A., Lawrence M.C. (1993). Sequence and structure alignment of paramyxovirus hemagglutinin-neuraminidase with influenza virus neuraminidase. J. Virol..

[83] WHO Summary of Neuraminidase (NA) Amino Acid Substitutions Assessed for Their Effects on Inhibition by NA Inhibitors (NAIs) Among Avian Influenza Viruses of Group 1 (N1, N4, N5, N8 subtypes) and Group 2 (N2, N3, N6, N7, N9 subtypes) NAs. https://cdn.who.int/media/docs/default-source/influenza/avwg/avian-nai-marker-who-table_07-08-2024_updated_final-version.pdf?sfvrsn=bc0d1e9a_5.

[84] Gubareva L.V., Robinson M.J., Bethell R.C., Webster R.G. (1997). Catalytic and framework mutations in the neuraminidase active site of influenza viruses that are resistant to 4-guanidino-Neu5Ac2en. J. Virol..

[85] Mishin V.P., Hayden F.G., Gubareva L.V. (2005). Susceptibilities of antiviral-resistant influenza viruses to novel neuraminidase inhibitors. Antimicrob. Agents Chemother..

[86] Kode S.S., Pawar S.D., Cherian S.S., Tare D.S., Bhoye D., Keng S.S., Mullick J. (2019). Selection of avian influenza A (H9N2) virus with reduced susceptibility to neuraminidase inhibitors oseltamivir and zanamivir. Virus Res..

[87] Tepper V., Nykvist M., Gillman A., Skog E., Wille M., Lindstrom H.S., Tang C., Lindberg R.H., Lundkvist A., Jarhult J.D. (2020). Influenza A/H4N2 mallard infection experiments further indicate zanamivir as less prone to induce environmental resistance development than oseltamivir. J. Gen. Virol..

[88] Gillman A., Muradrasoli S., Soderstrom H., Nordh J., Brojer C., Lindberg R.H., Latorre-Margalef N., Waldenstrom J., Olsen B., Jarhult J.D. (2013). Resistance mutation R292K is induced in influenza A(H6N2) virus by exposure of infected mallards to low levels of oseltamivir. PLoS ONE.

[89] Bialy D., Shelton H. (2020). Functional neuraminidase inhibitor resistance motifs in avian influenza A (H5Nx) viruses. Antivir. Res..

[90] Orozovic G., Orozovic K., Lennerstrand J., Olsen B. (2011). Detection of resistance mutations to antivirals oseltamivir and zanamivir in avian influenza A viruses isolated from wild birds. PLoS ONE.

[91] Song M.S., Marathe B.M., Kumar G., Wong S.S., Rubrum A., Zanin M., Choi Y.K., Webster R.G., Govorkova E.A., Webby R.J. (2015). Unique Determinants of Neuraminidase Inhibitor Resistance among N3, N7, and N9 Avian Influenza Viruses. J. Virol..

[92] Choi W.S., Jeong J.H., Kwon J.J., Ahn S.J., Lloren K.K.S., Kwon H.I., Chae H.B., Hwang J., Kim M.H., Kim C.J. (2017). Screening for Neuraminidase Inhibitor Resistance Markers among Avian Influenza Viruses of the N4, N5, N6, and N8 Neuraminidase Subtypes. J. Virol..

[93] Svyatchenko S.V., Goncharova N.I., Marchenko V.Y., Kolosova N.P., Shvalov A.N., Kovrizhkina V.L., Durymanov A.G., Onkhonova G.S., Tregubchak T.V., Susloparov I.M. (2021). An influenza A(H5N8) virus isolated during an outbreak at a poultry farm in Russia in 2017 has an N294S substitution in the neuraminidase and shows reduced susceptibility to oseltamivir. Antivir. Res..

[94] Xie R., Wang W., Gao Y., Liu W., Yue B., Liu S., Fan W., Song S., Yan L. (2023). Evolution and mammalian adaptation of H3 and H10 subtype avian influenza viruses in wild birds in Yancheng Wetland of China. Vet. Microbiol..

[95] Matsuoka Y., Swayne D.E., Thomas C., Rameix-Welti M.A., Naffakh N., Warnes C., Altholtz M., Donis R., Subbarao K. (2009). Neuraminidase stalk length and additional glycosylation of the hemagglutinin influence the virulence of influenza H5N1 viruses for mice. J. Virol..

[96] Li J., Zu Dohna H., Cardona C.J., Miller J., Carpenter T.E. (2011). Emergence and genetic variation of neuraminidase stalk deletions in avian influenza viruses. PLoS ONE.

[97] Munier S., Larcher T., Cormier-Aline F., Soubieux D., Su B., Guigand L., Labrosse B., Cherel Y., Quéré P., Marc D. (2010). A genetically engineered waterfowl influenza virus with a deletion in the stalk of the neuraminidase has increased virulence for chickens. J. Virol..

[98] Hoffmann T.W., Munier S., Larcher T., Soubieux D., Ledevin M., Esnault E., Tourdes A., Croville G., Guérin J.L., Quéré P. (2012). Length variations in the NA stalk of an H7N1 influenza virus have opposite effects on viral excretion in chickens and ducks. J. Virol..

[99] Bi Y., Xiao H., Chen Q., Wu Y., Fu L., Quan C., Wong G., Liu J., Haywood J., Liu Y. (2016). Changes in the Length of the Neuraminidase Stalk Region Impact H7N9 Virulence in Mice. J. Virol..

[100] Sorrell E.M., Song H., Pena L., Perez D.R. (2010). A 27-amino-acid deletion in the neuraminidase stalk supports replication of an avian H2N2 influenza A virus in the respiratory tract of chickens. J. Virol..

[101] Obenauer J.C., Denson J., Mehta P.K., Su X., Mukatira S., Finkelstein D.B., Xu X., Wang J., Ma J., Fan Y. (2006). Large-scale sequence analysis of avian influenza isolates. Science.

[102] Lan Y., Zhang Y., Dong L., Wang D., Huang W., Xin L., Yang L., Zhao X., Li Z., Wang W. (2010). A comprehensive surveillance of adamantane resistance among human influenza A virus isolated from mainland China between 1956 and 2009. Antivir. Ther..

[103] WHO Summary of Polymerase Acid Protein (PA) Amino Acid Substitutions for Their Effects on PA Inhibitor (PAI) Baloxavir Susceptibility. https://cdn.who.int/media/docs/default-source/influenza/laboratory---network/quality-assurance/antiviral-susceptibility-influenza/pa-marker-who-table_07-08-2024_updated_final-version.pdf?sfvrsn=5307d6fe_2.

[104] Andreev K., Jones J.C., Seiler P., Kandeil A., Webby R.J., Govorkova E.A. (2024). Genotypic and phenotypic susceptibility of emerging avian influenza A viruses to neuraminidase and cap-dependent endonuclease inhibitors. Antiviral Res..

[105] Xiao C., Ma W., Sun N., Huang L., Li Y., Zeng Z., Wen Y., Zhang Z., Li H., Li Q. (2016). PB2-588 V promotes the mammalian adaptation of H10N8, H7N9 and H9N2 avian influenza viruses. Sci. Rep..

[106] Hu M., Yuan S., Zhang K., Singh K., Ma Q., Zhou J., Chu H., Zheng B.J. (2017). PB2 substitutions V598T/I increase the virulence of H7N9 influenza A virus in mammals. Virology.

[107] Li J., Ishaq M., Prudence M., Xi X., Hu T., Liu Q., Guo D. (2009). Single mutation at the amino acid position 627 of PB2 that leads to increased virulence of an H5N1 avian influenza virus during adaptation in mice can be compensated by multiple mutations at other sites of PB2. Virus Res..

[108] Elgendy E.M., Arai Y., Kawashita N., Daidoji T., Takagi T., Ibrahim M.S., Nakaya T., Watanabe Y. (2017). Identification of polymerase gene mutations that affect viral replication in H5N1 influenza viruses isolated from pigeons. J. Gen. Virol..

[109] Feng X., Wang Z., Shi J., Deng G., Kong H., Tao S., Li C., Liu L., Guan Y., Chen H. (2015). Glycine at Position 622 in PB1 Contributes to the Virulence of H5N1 Avian Influenza Virus in Mice. J. Virol..

[110] Yamayoshi S., Yamada S., Fukuyama S., Murakami S., Zhao D., Uraki R., Watanabe T., Tomita Y., Macken C., Neumann G. (2014). Virulence-affecting amino acid changes in the PA protein of H7N9 influenza A viruses. J. Virol..

[111] Song J., Xu J., Shi J., Li Y., Chen H. (2015). Synergistic Effect of S224P and N383D Substitutions in the PA of H5N1 Avian Influenza Virus Contributes to Mammalian Adaptation. Sci. Rep..

[112] Tada T., Suzuki K., Sakurai Y., Kubo M., Okada H., Itoh T., Tsukamoto K. (2011). NP body domain and PB2 contribute to increased virulence of H5N1 highly pathogenic avian influenza viruses in chickens. J. Virol..

[113] Wasilenko J.L., Sarmento L., Pantin-Jackwood M.J. (2009). A single substitution in amino acid 184 of the NP protein alters the replication and pathogenicity of H5N1 avian influenza viruses in chickens. Arch. Virol..

[114] Fan S., Deng G., Song J., Tian G., Suo Y., Jiang Y., Guan Y., Bu Z., Kawaoka Y., Chen H. (2009). Two amino acid residues in the matrix protein M1 contribute to the virulence difference of H5N1 avian influenza viruses in mice. Virology.

[115] Nao N., Kajihara M., Manzoor R., Maruyama J., Yoshida R., Muramatsu M., Miyamoto H., Igarashi M., Eguchi N., Sato M. (2015). A Single Amino Acid in the M1 Protein Responsible for the Different Pathogenic Potentials of H5N1 Highly Pathogenic Avian Influenza Virus Strains. PLoS ONE.

[116] Jiao P., Tian G., Li Y., Deng G., Jiang Y., Liu C., Liu W., Bu Z., Kawaoka Y., Chen H. (2008). A single-amino-acid substitution in the NS1 protein changes the pathogenicity of H5N1 avian influenza viruses in mice. J. Virol..

[117] Li J., Zhang K., Chen Q., Zhang X., Sun Y., Bi Y., Zhang S., Gu J., Li J., Liu D. (2018). Three amino acid substitutions in the NS1 protein change the virus replication of H5N1 influenza virus in human cells. Virology.

[118] Kuo R.L., Krug R.M. (2009). Influenza a virus polymerase is an integral component of the CPSF30-NS1A protein complex in infected cells. J. Virol..

[119] Spesock A., Malur M., Hossain M., Chen L.M., Njaa B.L., Davis C.T., Lipatov A.S., York I.A., Krug R.M., Donis R.O. (2011). The virulence of 1997 H5N1 influenza viruses in the mouse model is increased by correcting a defect in their NS1 proteins. J. Virol..

[120] Takashita E., Morita H., Nagata S., Shirakura M., Fujisaki S., Miura H., Takayama I., Arita T., Suzuki Y., Yamaoka M. (2022). Influenza Virus Surveillance Group of Japan. Antiviral Susceptibilities of Avian Influenza A(H5), A(H7), and A(H9) Viruses Isolated in Japan. J. Infect. Dis..

[121] Liu K., Qi X., Bao C., Wang X., Liu X. (2024). Novel H10N3 avian influenza viruses: A potential threat to public health. Lancet Microbe.

[122] Bodewes R., Bestebroer T.M., van der Vries E., Verhagen J.H., Herfst S., Koopmans M.P., Fouchier R.A., Pfankuche V.M., Wohlsein P., Siebert U. (2015). Avian Influenza A(H10N7) virus-associated mass deaths among harbor seals. Emerg. Infect. Dis..

[123] De Marco M.A., Binazzi A., Melis P., Cotti C., Bonafede M., Delogu M., Tomao P., Vonesch N. (2025). Occupational Risk from Avian Influenza Viruses at Different Ecological Interfaces Between 1997 and 2019. Microorganisms.

[124] Zenatello M., Baccetti N., Borghesi F. Risulati dei Censimenti Degli Uccelli Acquatici Svernanti in Italia. Distribuzione, Stima e Trend Delle Popolazioni nel 2001–2010. ISPRA, Serie Rapporti, 206/2014. https://www.isprambiente.gov.it/files/pubblicazioni/rapporti/R_206_14_Uccelli_acquatici_svernanti_def.pdf.

[125] Brichetti P., Fracasso G. (2003). Germano Reale (*Anas platyrhynchos*). Ornitologia Italiana. Gavidae—Falconidae.

[126] Brichetti P., Fracasso G. (2004). Folaga (*Fulica atra*). Ornitologia Italiana. Tetraonidae—Scolopacidae.

[127] Kawaoka Y., Chambers T.M., Sladen W.L., Webster R.G. (1988). Is the gene pool of influenza viruses in shorebirds and gulls different from that in wild ducks?. Virology.

[128] McBride D.S., Lauterbach S.E., Li Y.T., Smith G.J.D., Killian M.L., Nolting J.M., Su Y.C.F., Bowman A.S. (2021). Genomic Evidence for Sequestration of Influenza A Virus Lineages in Sea Duck Host Species. Viruses.

[129] Hill N.J., Bishop M.A., Trovão N.S., Ineson K.M., Schaefer A.L., Puryear W.B., Zhou K., Foss A.D., Clark D.E., MacKenzie K.G. (2022). Ecological divergence of wild birds drives avian influenza spillover and global spread. PLoS Pathog..

[130] De Marco M.A., Foni E., Campitelli L., Raffini E., Delogu M., Donatelli I. (2003). Long-term monitoring for avian influenza viruses in wild bird species in Italy. Vet. Res. Commun..

[131] Matrosovich M.N., Matrosovich T.Y., Gray T., Roberts N.A., Klenk H.D. (2004). Human and avian influenza viruses target different cell types in cultures of human airway epithelium. Proc. Natl. Acad. Sci. USA.

[132] Hatta M., Gao P., Halfmann P., Kawaoka Y. (2001). Molecular basis for high virulence of Hong Kong H5N1 influenza A viruses. Science.

[133] Yang J., Yang L., Zhu W., Wang D., Shu Y. (2021). Epidemiological and Genetic Characteristics of the H3 Subtype Avian Influenza Viruses in China. China CDC Wkly..

[134] Long J.S., Howard W.A., Núñez A., Moncorgé O., Lycett S., Banks J., Barclay W.S. (2013). The effect of the PB2 mutation 627K on highly pathogenic H5N1 avian influenza virus is dependent on the virus lineage. J. Virol..

[135] Jones J.C., Yen H.L., Adams P., Armstrong K., Govorkova E.A. (2023). Influenza antivirals and their role in pandemic preparedness. Antivir. Res..

[136] Qi W., Zhou X., Shi W., Huang L., Xia W., Liu D., Li H., Chen S., Lei F., Cao L. (2014). Genesis of the novel human-infecting influenza A(H10N8) virus and potential genetic diversity of the virus in poultry, China. Eurosurveillance.

[137] WHO Summary of Neuraminidase (NA) Amino Acid Substitutions Assessed for Their Effects on Inhibition by Neuraminidase Inhibitors (NAIs). https://cdn.who.int/media/docs/default-source/influenza/laboratory---network/quality-assurance/human-nai-marker-table_for-publication_final_20240918.pdf?sfvrsn=c6d153ec_3.

[138] Taniguchi K., Noshi T., Omoto S., Sato A., Shishido T., Matsuno K., Okamatsu M., Krauss S., Webby R.J., Sakoda Y. (2024). The impact of PA/I38 substitutions and PA polymorphisms on the susceptibility of zoonotic influenza A viruses to baloxavir. Arch. Virol..

[139] Jetz W., Thomas G.H., Joy J.B., Hartmann K., Mooers A.O. (2012). The global diversity of birds in space and time. Nature.

[140] Elbe S., Buckland-Merrett G. (2017). Data, disease and diplomacy: GISAID’s innovative contribution to global health. Glob. Chall..

