# Supplementary material for: Divergent Avian Influenza H10 Viruses from Sympatric Waterbird Species in Italy: Zoonotic Potential Assessment by Molecular Markers
Source: Microorganisms. 2025 Nov 12;13(11):2575. doi: 10.3390/microorganisms13112575 (PMC12654176; doi:10.3390/microorganisms13112575)
Supplement: Supplementary file 1 [file microorganisms-13-02575-s001.zip › Figure S1.pdf]

	1	2	3	4	5	6	7	8	9		
**1**		99.0	83.9	84.3	84.2	83.9	84.2	83.9	83.6	**1**	A/Eurasian Coot/Italy/125/1994
**2**	1.0		84.6	84.9	84.8	84.5	84.8	84.3	84.0	**2**	A/Eurasian Coot/Italy/114/1995
**3**	18.8	17.8		97.5	97.3	97.1	97.3	94.3	94.4	**3**	A/Mallard/Italy/90/2002
**4**	18.3	17.5	2.5		99.2	99.0	99.2	93.6	93.9	**4**	A/Mallard/Italy/166998/2005
**5**	18.4	17.6	2.7	0.8		99.8	99.9	93.4	93.7	**5**	A/Mallard/Italy/Eco-634/2005
**6**	18.8	18.0	3.0	1.0	0.2		99.7	93.2	93.5	**6**	A/Mallard/Italy/Eco-7/2006
**7**	18.4	17.5	2.8	0.8	0.1	0.3		93.5	93.8	**7**	A/Mallard/Italy/Eco-33/2006
**8**	18.9	18.3	6.0	6.8	7.0	7.3	6.9		97.5	**8**	A/Mallard/Italy/Eco-360/2006
**9**	19.2	18.6	5.9	6.5	6.6	6.9	6.6	2.5		**9**	A/Mallard/Italy/195376/2007
	**1**	**2**	**3**	**4**	**5**	**6**	**7**	**8**	**9**		

HA percent similarity in upper triangle
HA percent divergence in lower triangle

Figure S1. HA genes similarity in avian H10NX strains under study.
