# Supplementary material for: Divergent Avian Influenza H10 Viruses from Sympatric Waterbird Species in Italy: Zoonotic Potential Assessment by Molecular Markers
Source: Microorganisms. 2025 Nov 12;13(11):2575. doi: 10.3390/microorganisms13112575 (PMC12654176; doi:10.3390/microorganisms13112575)
Supplement: Supplementary file 1 [file microorganisms-13-02575-s001.zip › Figure S2.pdf]

	1	2	3	4	5	6		
1		98.7	98.8	98.7	92.8	98.2	1	A/Mallard/Italy/166998/2005
2	1.3		99.9	99.9	92.7	98.7	2	A/Mallard/Italy/Eco-634/2005
3	1.2	0.1		99.9	92.7	98.7	3	A/Mallard/Italy/Eco-7/2006
4	1.3	0.1	0.1		92.7	98.7	4	A/Mallard/Italy/Eco-33/2006
5	7.7	7.9	7.8	7.9		92.4	5	A/Mallard/Italy/Eco-360/2006
6	1.8	1.4	1.3	1.4	8.1		6	A/Mallard/Italy/195376/2007
	1	2	3	4	5	6		

N7 Percent Similarity in upper triangle

N7 Percent Divergence in lower triangle

	1	2		
1		99.2	1	A/Eurasian Coot/Italy/114/1995
2	0.8		2	A/Eurasian Coot/Italy/125/1994
	1	2		

N8 Percent Similarity in upper triangle

N8 Percent Divergence in lower triangle

Figure S2. NA genes similarity in avian H10NX strains under study.
