# Supplementary material for: Divergent Avian Influenza H10 Viruses from Sympatric Waterbird Species in Italy: Zoonotic Potential Assessment by Molecular Markers
Source: Microorganisms. 2025 Nov 12;13(11):2575. doi: 10.3390/microorganisms13112575 (PMC12654176; doi:10.3390/microorganisms13112575)
Supplement: Supplementary file 1 [file microorganisms-13-02575-s001.zip › Figure S3.pdf]

| | 1 | 2 | 3 | 4 | 5 | 6 | 7 | 8 | 9 | | |
|---|---|---|---|---|---|---|---|---|---|---|---|
| **1** | | 99.5 | 89.3 | 89.9 | 89.7 | 89.7 | 89.7 | 88.9 | 89.9 | **1** | A/Eurasian Coot/Italy/125/1994 |
| **2** | 0.5 | | 89.4 | 89.7 | 89.5 | 89.5 | 89.5 | 88.5 | 89.7 | **2** | A/Eurasian Coot/Italy/114/1995 |
| **3** | 11.8 | 11.7 | | 93.7 | 93.3 | 93.3 | 93.5 | 96.7 | 93.9 | **3** | A/Mallard/Italy/90/2002 |
| **4** | 11.2 | 11.4 | 6.6 | | 99.3 | 99.3 | 99.3 | 93.1 | 98.9 | **4** | A/Mallard/Italy/166998/2005 |
| **5** | 11.4 | 11.7 | 7.2 | 0.7 | | 99.7 | 99.7 | 92.7 | 98.4 | **5** | A/Mallard/Italy/Eco-634/2005 |
| **6** | 11.3 | 11.6 | 7.1 | 0.7 | 0.3 | | 99.7 | 92.8 | 98.5 | **6** | A/Mallard/Italy/Eco-7/2006 |
| **7** | 11.3 | 11.6 | 6.9 | 0.7 | 0.3 | 0.3 | | 92.9 | 98.6 | **7** | A/Mallard/Italy/Eco-33/2006 |
| **8** | 12.4 | 12.8 | 3.4 | 7.4 | 7.8 | 7.7 | 7.6 | | 93.4 | **8** | A/Mallard/Italy/Eco-360/2006 |
| **9** | 11.1 | 11.3 | 6.5 | 1.1 | 1.6 | 1.6 | 1.4 | 7.0 | | **9** | A/Mallard/Italy/195376/2007 |
| | **1** | **2** | **3** | **4** | **5** | **6** | **7** | **8** | **9** | | |

**NP Percent Similarity in upper triangle**
**NP Percent Divergence in lower triangle**

Figure S3. NP genes similarity in avian H10NX strains under study.
