# Supplementary material for: Divergent Avian Influenza H10 Viruses from Sympatric Waterbird Species in Italy: Zoonotic Potential Assessment by Molecular Markers
Source: Microorganisms. 2025 Nov 12;13(11):2575. doi: 10.3390/microorganisms13112575 (PMC12654176; doi:10.3390/microorganisms13112575)
Supplement: Supplementary file 1 [file microorganisms-13-02575-s001.zip › Figure S4.pdf]

	1	2	3	4	5	6	7	8	9		
**1**		99.4	90.6	89.7	89.7	89.8	89.8	90.1	90.0	**1**	A/Eurasian Coot/Italy/125/1994
**2**	0.6		90.3	89.4	89.6	89.7	89.7	89.8	89.7	**2**	A/Eurasian Coot/Italy/114/1995
**3**	10.3	10.6		95.4	96.7	96.7	96.7	96.6	95.7	**3**	A/Mallard/Italy/90/2002
**4**	11.4	11.7	4.8		94.5	94.5	94.5	94.4	94.0	**4**	A/Mallard/Italy/166998/2005
**5**	11.3	11.5	3.4	5.8		100.0	99.9	95.5	94.9	**5**	A/Mallard/Italy/Eco-634/2005
**6**	11.3	11.4	3.4	5.8	0.0		100.0	95.6	94.9	**6**	A/Mallard/Italy/Eco-7/2006
**7**	11.2	11.3	3.4	5.8	0.1	0.0		95.5	94.9	**7**	A/Mallard/Italy/Eco-33/2006
**8**	10.9	11.2	3.5	6.0	4.7	4.6	4.7		94.8	**8**	A/Mallard/Italy/Eco-360/2006
**9**	11.0	11.3	4.5	6.4	5.3	5.3	5.3	5.4		**9**	A/Mallard/Italy/195376/2007
	**1**	**2**	**3**	**4**	**5**	**6**	**7**	**8**	**9**		

PA percent similarity in upper triangle
PA percent divergence in lower triangle

Figure S4. PA genes similarity in avian H10NX strains under study.
