# Supplementary material for: Divergent Avian Influenza H10 Viruses from Sympatric Waterbird Species in Italy: Zoonotic Potential Assessment by Molecular Markers
Source: Microorganisms. 2025 Nov 12;13(11):2575. doi: 10.3390/microorganisms13112575 (PMC12654176; doi:10.3390/microorganisms13112575)
Supplement: Supplementary file 1 [file microorganisms-13-02575-s001.zip › Figure S5.pdf]

	1	2	3	4	5	6	7	8	9		
**1**		99.1	91.1	91.3	91.3	91.3	91.4	91.1	91.3	**1**	A/Eurasian Coot/Italy/125/1994
**2**	0.9		90.7	91.1	91.1	91.1	91.2	90.9	91.0	**2**	A/Eurasian Coot/Italy/114/1995
**3**	9.7	10.2		94.0	94.0	94.2	94.1	94.2	94.0	**3**	A/Mallard/Italy/90/2002
**4**	9.5	9.7	6.3		99.3	99.3	99.4	97.5	97.5	**4**	A/Mallard/Italy/166998/2005
**5**	9.4	9.6	6.3	0.7		99.8	99.9	97.5	97.2	**5**	A/Mallard/Italy/Eco-634/2005
**6**	9.4	9.6	6.1	0.7	0.2		100.0	97.7	97.3	**6**	A/Mallard/Italy/Eco-7/2006
**7**	9.4	9.6	6.2	0.6	0.1	0.0		97.6	97.3	**7**	A/Mallard/Italy/Eco-33/2006
**8**	9.6	9.9	6.1	2.5	2.6	2.4	2.4		97.3	**8**	A/Mallard/Italy/Eco-360/2006
**9**	9.4	9.8	6.4	2.6	2.9	2.8	2.8	2.8		**9**	A/Mallard/Italy/195376/2007
	**1**	**2**	**3**	**4**	**5**	**6**	**7**	**8**	**9**		

PB1 percent similarity in upper triangle
PB1 percent divergence in lower triangle

Figure S5. PB1 genes similarity in avian H10NX strains under study.
