# Supplementary material for: Divergent Avian Influenza H10 Viruses from Sympatric Waterbird Species in Italy: Zoonotic Potential Assessment by Molecular Markers
Source: Microorganisms. 2025 Nov 12;13(11):2575. doi: 10.3390/microorganisms13112575 (PMC12654176; doi:10.3390/microorganisms13112575)
Supplement: Supplementary file 1 [file microorganisms-13-02575-s001.zip › Figure S6.pdf]

	1	2	3	4	5	6	7	8	9		
**1**		99.3	88.6	88.3	87.9	87.8	87.8	88.3	88.0	**1**	A/Eurasian Coot/Italy/125/1994
**2**	0.7		88.6	88.2	87.8	87.7	87.7	88.3	87.9	**2**	A/Eurasian Coot/Italy/114/1995
**3**	12.7	12.7		96.1	95.0	95.0	95.0	95.5	94.9	**3**	A/Mallard/Italy/90/2002
**4**	13.1	13.3	4.0		94.8	94.7	94.8	94.9	94.5	**4**	A/Mallard/Italy/166998/2005
**5**	13.7	13.8	5.2	5.4		99.9	100.0	96.1	98.1	**5**	A/Mallard/Italy/Eco-634/2005
**6**	13.8	13.9	5.3	5.5	0.1		100.0	96.1	98.0	**6**	A/Mallard/Italy/Eco-7/2006
**7**	13.7	13.8	5.2	5.5	0.0	0.0		96.1	98.0	**7**	A/Mallard/Italy/Eco-33/2006
**8**	13.1	13.0	4.7	5.3	4.0	4.1	4.1		96.0	**8**	A/Mallard/Italy/Eco-360/2006
**9**	13.4	13.5	5.3	5.8	2.0	2.1	2.0	4.2		**9**	A/Mallard/Italy/195376/2007
	**1**	**2**	**3**	**4**	**5**	**6**	**7**	**8**	**9**		

PB2 percent similarity in upper triangle
PB2 percent divergence in lower triangle

Figure S6. PB2 genes similarity in avian H10NX strains under study.
