# Supplementary material for: Divergent Avian Influenza H10 Viruses from Sympatric Waterbird Species in Italy: Zoonotic Potential Assessment by Molecular Markers
Source: Microorganisms. 2025 Nov 12;13(11):2575. doi: 10.3390/microorganisms13112575 (PMC12654176; doi:10.3390/microorganisms13112575)
Supplement: Supplementary file 1 [file microorganisms-13-02575-s001.zip › Figure S7.pdf]

	1	2	3	4	5	6	7	8	9		
1		99.7	96.7	95.8	97.0	96.9	96.9	96.5	96.5	1	A/Eurasian Coot/Italy/125/1994
2	0.3		96.5	95.6	96.9	96.8	96.8	96.5	96.3	2	A/Eurasian Coot/Italy/114/1995
3	3.4	3.6		97.9	96.4	96.3	96.3	98.0	97.6	3	A/Mallard/Italy/90/2002
4	4.4	4.6	2.2		95.9	95.8	95.8	97.3	97.3	4	A/Mallard/Italy/166998/2005
5	3.1	3.2	3.7	4.3		99.9	99.9	96.0	96.3	5	A/Mallard/Italy/Eco-634/2005
6	3.2	3.3	3.8	4.4	0.1		99.8	95.9	96.2	6	A/Mallard/Italy/Eco-7/2006
7	3.2	3.3	3.8	4.4	0.1	0.2		95.9	96.2	7	A/Mallard/Italy/Eco-33/2006
8	3.6	3.6	2.0	2.8	4.2	4.3	4.3		97.8	8	A/Mallard/Italy/Eco-360/2006
9	3.6	3.9	2.5	2.8	3.9	4.0	4.0	2.3		9	A/Mallard/Italy/195376/2007
	1	2	3	4	5	6	7	8	9		

MP percent similarity in upper triangle
MP percent divergence in lower triangle

Figure S7. MP genes similarity in avian H10NX strains under study.
