# Supplementary material for: Divergent Avian Influenza H10 Viruses from Sympatric Waterbird Species in Italy: Zoonotic Potential Assessment by Molecular Markers
Source: Microorganisms. 2025 Nov 12;13(11):2575. doi: 10.3390/microorganisms13112575 (PMC12654176; doi:10.3390/microorganisms13112575)
Supplement: Supplementary file 1 [file microorganisms-13-02575-s001.zip › Figure S8.pdf]

	1	2	3	4	5	6	7	8	9		
1		99.3	92.5	91.8	92.2	92.1	92.2	92.0	72.0	1	A/Eurasian Coot/Italy/125/1994
2	0.7		92.5	91.8	92.2	92.1	92.2	92.0	72.4	2	A/Eurasian Coot/Italy/114/1995
3	8.0	8.0		98.7	99.2	99.1	99.2	98.2	71.8	3	A/Mallard/Italy/90/2002
4	8.8	8.8	1.3		98.8	98.7	98.8	97.9	71.7	4	A/Mallard/Italy/166998/2005
5	8.3	8.3	0.8	1.2		99.6	99.8	98.6	71.8	5	A/Mallard/Italy/Eco-634/2005
6	8.4	8.4	0.9	1.3	0.4		99.9	98.5	71.8	6	A/Mallard/Italy/Eco-7/2006
7	8.3	8.3	0.8	1.2	0.2	0.1		98.6	71.9	7	A/Mallard/Italy/Eco-33/2006
8	8.6	8.6	1.8	2.2	1.4	1.5	1.4		72.0	8	A/Mallard/Italy/Eco-360/2006
9	35.1	34.6	35.5	35.7	35.5	35.5	35.3	35.1		9	A/Mallard/Italy/195376/2007
	1	2	3	4	5	6	7	8	9		

NS percent similarity in upper triangle

NS percent divergence in lower triangle

Figure S8. NS genes similarity in avian H10NX strains under study.
