# Supplementary material for: Divergent Avian Influenza H10 Viruses from Sympatric Waterbird Species in Italy: Zoonotic Potential Assessment by Molecular Markers
Source: Microorganisms. 2025 Nov 12;13(11):2575. doi: 10.3390/microorganisms13112575 (PMC12654176; doi:10.3390/microorganisms13112575)
Supplement: Supplementary file 1 [file microorganisms-13-02575-s001.zip › Table S1.pdf]

Table S1: Summary of HA mutations screened in the nine H10 AIV strains under study (Italy 1994-2007) and known to be associated with zoonotic potential, as previously reviewed [36].

Subtype tested	Amino acid change/Motif (H3 numbering)	Phenotypic conseguences	References
H5N1	D101N	Increased virus binding to α2–6	[38]
	S126N	Increased virus binding to α2–6	[39]
	S137A	Increased pseudovirus binding to α2–6	[40]
	A138V	Increased infectivity in SIATCells	[41,42]
	G143R	Increased virus binding to α2–6	[43]
	S158N	Increased virus binding to α2–6	[39]
	S159N	Increased virus binding to α2–6	[39]
	T160A	Increased virus binding to α2–6, increased transmission in guinea pigs	[39,44]
	N186K/D	Increased virus binding to α2–6	[39]
	D187G	Increased virus binding to α2–6	[45]
	E190G	Increased virus binding to α2–6, maintained α2–3 binding, decreased virulence in mice	[45,46]
	T192I	Increased pseudovirus binding to α2–6	[43]
	K193R/T	Increased virus binding to α2–6	[39]
	Q196R/H	Increased virus binding to α2–6	[45]
	N197K	Increased virus binding to α2–6	[43]
	V214I	Increased virus binding to α2–6	[47]
	S221P	Increased virus binding to α2–6	[48]

	S221P+K216E	Increased virus binding to α2–6	[48]
	Q226L	Increased virus binding to α2–6, decreased binding to α2–3	[49]
	S227N	Increased virus binding to α2–6	[49]
	G228S	Increased virus binding to α2–6	[50]
	E83K+S128P	Increased virus binding to α2–6	[43]
	E83K+S128P+R496K	Increased virus binding to α2–6	[43]
	E83K+S128P+N197K+R496K	Increased virus binding to α2–6	[43]
	E83K+N197K	Increased virus binding to α2–6	[43]
	E83K+N197K+R496K	Increased virus binding to α2–6	[43]
	E83K+R496K	Increased virus binding to α2–6	[43]
	H110Y+T160A+Q226L+G228S	Airborne transmissible in ferrets,	[51,52]
	S114R+T115I	Increased virulence in chickens and mice, increased pH of fusion	[53]
	S128P+N197K	Increased virus binding to α2–6	[43]
	S128P+N197K+R496K	Increased virus binding to α2–6	[43]
	S128P+R496K	Increased virus binding to α2–6	[43]
	P239S	Increased virus binding to α2–6	[43]
	E255K	Increased virus binding to α2–6	[45]
	S137A+T192I	Increased pseudovirus binding to α2–6	[43]
	G143R+N186K	Decreased binding to α2–3, increased virus binding to α2–6	[49]
	K387I	Decreased pH of fusion, increased HA stability, increased replication, decreased virulence in mice, efficiency and virulence in mice	[54,55,56]
	N158S+Q226L	Increased virus binding to α2–6	[57]
	N158S+Q226L+N248D	Increased virus binding to α2–6	[57]

**H5N1**	S159N+T160A	Increased virus binding to α2–6	[39]
	S159N+T160A+S227N	Increased virus binding to α2–6, reduced lethality and systemic spread in mice	[58]
	T160A+K193T+N224K+Q226L	Increased virus binding to α2–6	[59]
	T160A+Q226L	Increased virus binding to α2–6	[39]
	T160A+Q226L+G228S	Increased virus binding to α2–6	[39]
	T160A+S227N	Increased virus binding to α2–6	[39]
	N186K+Q226L+S227N+G228S	Increased virus binding to α2–6	[49]
	N186K+Q226L+G228S	Increased virus binding to α2–6	[49]
	E187G+E190D+K193S+Q226L+G228S	Increased virus binding to α2–6	[60]
	E187G+Q226L+G228S	Increased virus binding to α2–6	[45]
	D187G+S227N	Increased virus binding to α2–6	[45]
	N186K+Q196R+Q226L+S227N+G228S	Increased virus binding to α2–6	[49]
	E190G+Q226E+G228S	Increased virus binding to α2–6	[45]
	K193R+Q226L+G228S	Increased virus binding to α2–6	[59]
	Q196R+Q226L+S227N+G228S	Increased virus binding to α2–6	[49]
	Q196R+Q226L+G228S	Increased virus binding to α2–6	[49]
	Q196R+S227N	Increased virus binding to α2–6	[49]
	N197K+R496K	Increased virus binding to α2–6	[43]
	K222Q+S227R	Increased virus binding to α2–3 and α2–6	[61]
	Q226L+S227N+G228S	Increased virus binding to α2–6	[49]
	Q226L+G228S	Increased virus binding to α2–6; decreased antiviral response in host; reduced tissue tropism in guinea pigs	[45,49,50]
	326 to 329 (R-X-R K-R)	Polybasic cleavage motif sequence required for high pathogenicity avian influenza viruses	[62]

	N158D+N224K+Q226L+T318I	Transmissible among ferrets	[63]
**H7N9**	G186V	Increased virus binding to α2–3	[64]
	Q226L	Increased virus binding to α2–3, decreased binding to α2–6	[65,66]
	L226I	Decreased binding to α2–3	[65]
	G228S	Decreased binding to α2–3 and α2–6 receptors	[67]
	K393E	Increased pH of fusion, decreased HA stability	[36,37]
	V186N, N228K	Increased virus binding to α2–6	[67]
	V186K/G+K193T+G228S	Increased virus binding to α2–6,	[67]
	V186N+N224K+G228S	Increased virus binding to α2–6,	[67]
	K193T+G228S	Dual α2–3 and α2–6 binding	[67]
	N224K+G228S	Increased virus binding to α2–6	[67]
**H7N7**	Q226L+G228S	Increased virus binding to α2–6	[68]
**H9N2**	N158D	Decreased virulence in mice	[69]
	T190V	Enhances binding affinity to mammalian cells and replicationin mammalian cells	[70]
	Q226L	Increased virus binding to α2–6, enhanced replication in mammalian cells and in ferrets, enhanced contact transmission in ferrets	[71,72]
	T189A+G192R	Enhanced replication in ferrets, transmitted via aerosols among ferrets,	[73]

**H6N1**	P186L	Decreased binding to α2–3	[74]
	G225D	Increased virus binding to α2–6	[75]
**H6N2**	E190V	Decreased binding to α2–3 and α2–6	[76]
	Q226L	Increased virus binding to α2–6	[76]
	G228S	Decreased virus binding to α2–3	[76]
**H4N6**	Q226L	Increased virus binding to α2–6	[77]
	G228A/S	Increased binding to α2–6, dual receptor specificity	[77]
	Q226L+G228S	Increased virus binding to α2–6	[77]
**H1N1**	G228S	Increased viral replication in mammalian cells and virulence in mice	[78]
**H10N8**	Q226L	Loss of binding to α2–3	[79,80]
	G228S	Decreased binding to α2–3, no binding to α2–6	[80]
	Q226L+G228S	Loss of binding to α2–3, no gain of binding to α2–6,	[79,80]
**H13N6**	V186N	Increased binding to α2–6, decreased binding to α2–3	[81]
