# Supplementary material for: Divergent Avian Influenza H10 Viruses from Sympatric Waterbird Species in Italy: Zoonotic Potential Assessment by Molecular Markers
Source: Microorganisms. 2025 Nov 12;13(11):2575. doi: 10.3390/microorganisms13112575 (PMC12654176; doi:10.3390/microorganisms13112575)
Supplement: Supplementary file 1 [file microorganisms-13-02575-s001.zip › Table S2.pdf]

Table S2. Summary of N2, N7 and N8 mutations screened in the nine H10 AIV strains under study (Italy 1994-2007) and known to be associated with reduced susceptibility to oseltamivir and zanamivir drugs, as previously reviewed [36, 83].

N Subtypes	Amino acid change (N2 numbering)	Phenotypic conseguences	References
**N2**	E119A	Reduced susceptibility to zanamivir	[84,85]
	E119D	Reduced susceptibility to zanamivir	[84,86]
	E119G	Reduced susceptibility to zanamivir	[84,87]
	R292K	Reduced susceptibility to oseltamivir and zanamivir	[84,86,88]
	N294S	Reduced susceptibility to zanamivir	[89]
	I314V	Reduced susceptibility to oseltamivir	[90]
**N7**	E119G	Reduced susceptibility to zanamivir	[91]
	E119D	Reduced susceptibility to zanamivir	[91]
	E119V	Reduced susceptibility to oseltamivir	[91]
	R152W	Reduced susceptibility to oseltamivir and zanamivir	[91]
	H274Y	Reduced susceptibility to oseltamivir	[91]
	E276D	Reduced susceptibility to zanamivir	[91]
	R292K	Reduced susceptibility to oseltamivir and zanamivir	[91]
	D293N	Reduced susceptibility to oseltamivir and zanamivir	[91]
	S111N+E119D	Reduced susceptibility to zanamivir	[91]
	I275T+E276D	Reduced susceptibility to zanamivir	[91]
**N8**	E119V	Reduced susceptibility to oseltamivir and zanamivir	[89]
	Q136K	Reduced susceptibility to zanamivir	[92]
	G147V	Reduced susceptibility to zanamivir	[92]
	H274Y	Reduced susceptibility to oseltamivir	[89]
	R292K	Reduced susceptibility to oseltamivir and zanamivir	[92]
	N294S	Reduced susceptibility to oseltamivir	[89,93]
