# Supplementary material for: Divergent Avian Influenza H10 Viruses from Sympatric Waterbird Species in Italy: Zoonotic Potential Assessment by Molecular Markers
Source: Microorganisms. 2025 Nov 12;13(11):2575. doi: 10.3390/microorganisms13112575 (PMC12654176; doi:10.3390/microorganisms13112575)
Supplement: Supplementary file 1 [file microorganisms-13-02575-s001.zip › Table S3.pdf]

Table S3. Summary of NA mutations screened in in the nine H10 AIV strains under study (Italy 1994-2007) and known to be associated to zoonotic potential, as previously reported [36].

N Subtypes	Amino acid change (N2 numbering)	Phenotypic conseguences	References
N1	49–68 deletion	Enhanced virulence in mice	[95]
	54–72 deletion	Enhanced virulence in mice but not chickens	[96]
	54–72 deletion	Enhanced virulence in mice and chickens	[97]
	54–75 deletion	Enhanced virulence in chickens but not ducks	[98]
N9	54–72 deletion	Enhanced virulence in mice	[99]
	54–73 deletion	Enhanced virulence in mice	[99]
	57–65 deletion	Enhanced virulence in mice	[99]
N2	54–81 deletion	Enhanced replication in chicken cell line. Enhanced replication in respiratory tract of chickens.	[100]
