# Supplementary material for: Divergent Avian Influenza H10 Viruses from Sympatric Waterbird Species in Italy: Zoonotic Potential Assessment by Molecular Markers
Source: Microorganisms. 2025 Nov 12;13(11):2575. doi: 10.3390/microorganisms13112575 (PMC12654176; doi:10.3390/microorganisms13112575)
Supplement: Supplementary file 1 [file microorganisms-13-02575-s001.zip › Table S4.pdf]

Table S4. Multiple mutations detected in internal protein genes of the nine H10NX AIVs under study. In light blue cells the amino acid substitutions previously reported to be linked to increased replication in mammalian and avian cells, and to increased virulence in mice, ducks, chickens and pigs.

Viruses	PB2					
	R340K	K389R	A588V	V598T	L89V+G309D	L89V+G309D+T339K+R477G+I495V +K627E+A676T
**A/Eurasian Coot/It/125/1994** (H10N8)	K	R	V	T	V+D	V+D+K+G+V+E+T
**A/Eurasian Coot/It/114/1995** (H10N8)	K	R	V	T	V+D	V+D+K+G+V+E+T
**A/Mallard/It/90/2002** (H10N2)	R	R	A	T	V+D	V+D+K+G+V+E+T
**A/Mallard/It/166998/2005** (H10N7)	R	R	A	T	V+D	V+D+K+G+V+E+T
**A/Mallard/It/Eco-634/2005** (H10N7)	K	R	A	T	V+D	V+D+K+G+V+E+T
**A/Mallard/It/Eco-7/2006** (H10N7)	K	R	A	T	V+D	V+D+K+G+V+E+T
**A/Mallard/It/Eco-33/2006** (H10N7)	K	R	A	T	V+D	V+D+K+G+V+E+T
**A/Mallard/It/Eco-360/2006** (H10N7)	R	R	A	T	V+D	V+D+K+G+V+E+T
**A/Mallard/It/195376/2007** (H10N7)	R	R	A	T	V+D	V+D+K+G+V+E+T

Viruses	PB1		PA			NP		
	D3V	D622G	S37A	N383D	N409S	M105V	I109T	A184K
**A/Eurasian Coot/It/125/1994** (H10N8)	V	G	A	D	S	M	I	K
**A/Eurasian Coot/It/114/1995** (H10N8)	V	G	A	D	S	M	I	K
**A/Mallard/It/90/2002** (H10N2)	V	G	A	D	S	M	I	K
**A/Mallard/It/166998/2005** (H10N7)	V	G	A	D	S	V	I	K
**A/Mallard/It/Eco-634/2005** (H10N7)	V	G	A	D	S	V	I	K
**A/Mallard/It/Eco-7/2006** (H10N7)	V	G	A	D	S	V	I	K
**A/Mallard/It/Eco-33/2006** (H10N7)	V	G	A	D	S	V	T	K
**A/Mallard/It/Eco-360/2006** (H10N7)	V	G	A	D	S	V	I	K
**A/Mallard/It/195376/2007** (H10N7)	V	G	A	D	S	V	I	K

Viruses	M1			NS1				
	N30D	I43M	T215A	P42S	C138F	V149A	L103F + I106M	K55E + K66E + C138F
**A/Eurasian Coot/It/125/1994** (H10N8)	D	M	A	S	F	A	F + M	E + E + F
**A/Eurasian Coot/It/114/1995** (H10N8)	D	M	A	S	F	A	F + M	E + E + F
**A/Mallard/It/90/2002** (H10N2)	D	M	A	S	F	A	F + M	E + E + F
**A/Mallard/It/166998/2005** (H10N7)	D	M	A	S	F	A	F + M	E + E + F
**A/Mallard/It/Eco-634/2005** (H10N7)	D	M	A	S	F	A	F + M	E + E + F
**A/Mallard/It/Eco-7/2006** (H10N7)	D	M	A	S	F	A	F + M	E + E + F
**A/Mallard/It/Eco-33/2006** (H10N7)	D	M	A	S	F	A	F + M	E + E + F
**A/Mallard/It/Eco-360/2006** (H10N7)	D	M	A	S	F	A	F + M	E + E + F
**A/Mallard/It/195376/2007** (H10N7)	D	M	A	A	F	A	Y + M	R + E + F
