# Supplementary material for: Divergent Avian Influenza H10 Viruses from Sympatric Waterbird Species in Italy: Zoonotic Potential Assessment by Molecular Markers
Source: Microorganisms. 2025 Nov 12;13(11):2575. doi: 10.3390/microorganisms13112575 (PMC12654176; doi:10.3390/microorganisms13112575)
Supplement: Supplementary file 1 [file microorganisms-13-02575-s001.zip › Table S5.pdf]

Table S5. GISAID accession numbers of HA sequences used in the study.

Isolate_Id	HA Segment_Id	Isolate_Name
EPI_ISL_30000	EPI178543	A/Quail/Italy/1966
EPI_ISL_89161	EPI314135	A/quail/Italy/1117/1965
EPI_ISL_10065	EPI101951	A/duck/IT/701/2005
EPI_ISL_29991	EPI178493	A/Duck/Italy/268302/2004
EPI_ISL_29228	EPI174777	A/duck/Italy/73383/2006
EPI_ISL_29998	EPI178528	A/Duck/Italy/62330/2006
EPI_ISL_29999	EPI178536	A/Duck/Italy/60772/2007
EPI_ISL_158687	EPI511811	A/mallard/Italy/4518/2007
EPI_ISL_30001	EPI178551	A/Mallard/Italy/46341-12/2006
EPI_ISL_69928	EPI242465	A/quail/Italy/39403/1965
EPI_ISL_29971	EPI178459	A/Turkey/Italy/928/1967
EPI_ISL_19213564	EPI3394246	A/duck/Italy/4481/2014
EPI_ISL_73376	EPI251700	A/mallard/Sweden/4/2002
EPI_ISL_189470	EPI618122	A/mallard/Sweden/2823/2003
EPI_ISL_189349	EPI618970	A/mallard/Sweden/3151/2003
EPI_ISL_189352	EPI619009	A/mallard/Sweden/4411/2004
EPI_ISL_189350	EPI618984	A/mallard/Sweden/4258/2004
EPI_ISL_189471	EPI618130	A/mallard/Sweden/6039/2005
EPI_ISL_189354	EPI618868	A/mallard/Sweden/5824/2005
EPI_ISL_189366	EPI618764	A/mallard/Sweden/52903/2006
EPI_ISL_189368	EPI618792	A/mallard/Sweden/64476/2007
EPI_ISL_68416	EPI238130	A/mallard/Netherlands/02/2000
EPI_ISL_267274	EPI1010683	A/mallard duck/Netherlands/7/2006
EPI_ISL_84566	EPI296584	A/mute swan/Netherlands/1/2006
EPI_ISL_84565	EPI296576	A/herring gull/Netherlands/4/2006
EPI_ISL_243388	EPI889847	A/mallard duck/Netherlands/1/2007
EPI_ISL_10383	EPI164597	A/mallard/Bavaria/3/2006
EPI_ISL_120212	EPI372496	A/pied avocet/Ukraine/05848-NAMRU3/2006
EPI_ISL_611	EPI3085	A/duck/Hong Kong/786/1979
EPI_ISL_70091	EPI243044	A/chicken/Germany/N/1949
EPI_ISL_410	EPI1948	A/duck/Hong Kong/938/80
EPI_ISL_209136	EPI704454	A/duck/Hong Kong/934/1980
EPI_ISL_594	EPI3013	A/swan/Shimane/1331/1981
EPI_ISL_89934	EPI407701	A/chicken/Hubei/119/1983
EPI_ISL_132818	EPI407688	A/duck/Hubei/137/1985
EPI_ISL_26348	EPI169485	A/Mallard/Gloucestershire/PD374/1985
EPI_ISL_279426	EPI1067689	A/mandarin duck/Singapore/805_F-72_7/1993
EPI_ISL_631	EPI3199	A/duck/Shimane/45/1997
EPI_ISL_497	EPI2258	A/duck/Hokkaido/18/00
EPI_ISL_157901	EPI510260	A/Eurasian coot/Germany/R411/2010
EPI_ISL_120189	EPI372315	A/shoveler/Egypt/09782-NAMRU3/2004
EPI_ISL_252870	EPI942341	A/duck/Shanghai/602/2009
EPI_ISL_309179	EPI1226308	A/duck/Bangladesh/821/2009
EPI_ISL_252872	EPI942343	A/duck/Shanxi/3180/2010
EPI_ISL_309839	EPI1230143	A/Chicken/Netherlands/09007064/2009
EPI_ISL_257184	EPI966038	A/turkey/Netherlands/09006938/2009
EPI_ISL_181981	EPI600909	A/migratory duck/Jiangxi/10861/2005
EPI_ISL_181999	EPI600845	A/migratory duck/Jiangxi/593/2005
EPI_ISL_182001	EPI600858	A/duck/Jiangxi/4759/2009
EPI_ISL_397992	EPI1625235	A/ruddy turnstone/New Jersey/471614/2001
EPI_ISL_180862	EPI595360	A/mallard/Maryland/06OS2409/2006
EPI_ISL_138173	EPI438120	A/mallard/Alberta/57/2004

EPI_ISL_8891	EPI90298	A/blue-winged teal/ALB/778/1978
EPI_ISL_189392	EPI618608	A/mallard/Sweden/133546/2011
EPI_ISL_169276	EPI552753	A/mallard/Netherlands/47/2010
EPI_ISL_166243	EPI541472	A/mallard/Denmark/16109-4/2011-11-14
EPI_ISL_243453	EPI889848	A/mallard duck/Netherlands/31/2013
EPI_ISL_169274	EPI552751	A/mallard/Netherlands/1/2014
EPI_ISL_90293	EPI317614	A/Mallard/Germany/R2075/2007
EPI_ISL_189348	EPI618957	A/mallard/Sweden/1417/2002
EPI_ISL_19699133	EPI3867764	A/Mallard/Sweden/SVA241211SZ0514/FB284289/OT/2024
EPI_ISL_19700676	EPI3873319	A/Mallard/Sweden/SVA241211SZ0644/FB285298/OT/2024
EPI_ISL_19634850	EPI3741763	A/Mallard/Sweden/SVA241126SZ0424/FB268641/Ot/H/2024
EPI_ISL_19277004	EPI3447895	A/common_murre/Spain/1034-2_24VIR4860-21/2024
EPI_ISL_18343240	EPI2764128	A/Anas_platyrhynchos/Belgium/00366_0008/2023
EPI_ISL_19363646	EPI3523279	A/Anas_platyrhynchos/Belgium/01235_0002/2024
EPI_ISL_201998	EPI709089	A/Seal/Sweden/SVA1412040224-SZ1847/H10N7/2014
EPI_ISL_202002	EPI709101	A/harbor_seal/NL/PV325-14_NS/NL/2014
EPI_ISL_202018	EPI709117	A/harbor_seal/GER/S1070_14_Tr/2014
EPI_ISL_202030	EPI709129	A/harbor_seal/GER/S1047_14_L/2014
EPI_ISL_202043	EPI709142	A/harbor_seal/GER/PV20766_Tr/2014
EPI_ISL_166971	EPI544351	A/harbour_seal/Germany/1/2014
EPI_ISL_167225	EPI545210	A/Seal/Sweden/SVA0546/2014
EPI_ISL_202050	EPI709143	A/harbor_seal/DK/14-8148_L/2014
EPI_ISL_202055	EPI709090	A/Seal/Sweden/SVA1412040224-SZ5634/H10N7/2014
EPI_ISL_166244	EPI541474	A/harbor_seal/Denmark/14-5061-1lu/2014-07
EPI_ISL_13482654	EPI2076241	A/harbor_seal/British_Colombia/OTH-52-1/2021
EPI_ISL_68973	EPI240179	A/mink/Sweden/1984
EPI_ISL_26266	EPI169313	A/Mink/Sweden/3900/1984
EPI_ISL_129071	EPI395080	A/swine/Hubei/10/2008
EPI_ISL_1005678	EPI1846408	A/common teal/Chany Lake/4/2020
EPI_ISL_14388281	EPI2122801	A/Cygnus_olor/Belgium/11638_0002/2021
EPI_ISL_14393464	EPI2122964	A/Arenaria_interpres/Belgium/3125_0002/2022
EPI_ISL_189392	EPI618608	A/mallard/Sweden/133546/2011
EPI_ISL_309809	EPI1229914	A/Chicken/Netherlands/12002495-001-005/2012
EPI_ISL_502609	EPI1774464	A/Anas platyrhynchos/Belgium/398_H189467/2017
EPI_ISL_169274	EPI552751	A/mallard/Netherlands/1/2014
EPI_ISL_18794371	EPI2934202	A/mallard/Dagestan/004/2018
EPI_ISL_309174	EPI1226337	A/duck/Bangladesh/24268/2015
EPI_ISL_256785	EPI963336	A/duck/Bangladesh/24035/2014
EPI_ISL_173536	EPI599684	A/chicken/Jiangxi/1204/2014
EPI_ISL_273604	EPI1072992	A/American black duck/Alberta/274/2016
EPI_ISL_66123	EPI230698	A/mallard/California/K752/2006
EPI_ISL_180862	EPI595360	A/mallard/Maryland/06OS2409/2006
EPI_ISL_138173	EPI438120	A/mallard/Alberta/57/2004
EPI_ISL_279425	EPI1067688	A/turkey/Minnesota/5/1979
EPI_ISL_5835	EPI42397	A/mallard duck/Minnesota/19/1979
EPI_ISL_78790	EPI277555	A/mallard/Sweden/51/2002
EPI_ISL_189356	EPI618898	A/mallard/Sweden/6148/2005
EPI_ISL_189366	EPI618764	A/mallard/Sweden/52903/2006
EPI_ISL_189359	EPI618863	A/mallard/Sweden/51582/2006
EPI_ISL_189370	EPI618823	A/mallard/Sweden/69777/2007
EPI_ISL_189367	EPI618778	A/mallard/Sweden/59463/2007
EPI_ISL_267318	EPI1014139	A/mallard duck/Netherlands/2/2014
EPI_ISL_243574	EPI890279	A/mallard duck/Netherlands/2/2011
EPI_ISL_243534	EPI890897	A/mallard duck/Netherlands/1/2012
EPI_ISL_243491	EPI889579	A/mallard duck/Netherlands/32/2013

EPI_ISL_243393	EPI890232	A/mallard duck/Netherlands/6/2015
EPI_ISL_379990	EPI1557401	A/Iceland gull/Iceland/4402/2015
EPI_ISL_379992	EPI1557417	A/glaucous gull/Iceland/4552/2015
EPI_ISL_166243	EPI541472	A/mallard/Denmark/16109-4/2011-11-14
EPI_ISL_502606	EPI1774408	A/Anas platyrhynchos/Belgium/1837_H101620/2018
EPI_ISL_26346	EPI169469	A/WhistlingSwan/Shimane/468/1988
EPI_ISL_399746	EPI1635105	A/duck/Cambodia/C8W6M1/2018
EPI_ISL_390	EPI161540	A/duck/Mongolia/149/03
EPI_ISL_504329	EPI1780768	A/duck/Shimane/321103/2010
EPI_ISL_162077	EPI530542	A/chicken/Jiangxi/102/2013(H10N8)
EPI_ISL_181981	EPI600909	A/migratory duck/Jiangxi/10861/2005
EPI_ISL_125924	EPI387870	A/mallard/Korea/1242/2010
EPI_ISL_181615	EPI600178	A/migratory duck/Jiangxi/33238/2013
EPI_ISL_505078	EPI1785771	A/wild birds/Hubei/102/2014
EPI_ISL_182001	EPI600858	A/duck/Jiangxi/4759/2009
EPI_ISL_309178	EPI1226298	A/chicken/Bangladesh/842/2009
EPI372481	EPI_ISL_120210	A/teal/Egypt/12908-NAMRU3/2005
EPI456976	EPI_ISL_138759	A/avian/Israel/824/2005
EPI1175838	EPI_ISL_298536	A/duck/Mongolia/493/2010
EPI600867	EPI_ISL_182002	A/migratory duck/Jiangxi/21248/2009
EPI387868	EPI_ISL_125923	A/wild bird/Korea/A12/2010
EPI_ISL_617	EPI3113	A/duck/Hong Kong/562/1979
EPI3725083	EPI_ISL_19613962	A/Mallard_Duck/Netherlands/34/2018
EPI3725115	EPI_ISL_19613966	A/Mallard_Duck/Netherlands/38/2018
EPI3724915	EPI_ISL_19613941	A/Mallard_Duck/Netherlands/51/2018
EPI461563	EPI_ISL_143931	A/duck/Hunan/S11205/2012
EPI438281	EPI_ISL_138236	A/duck/Vietnam/LBM300/2012
EPI387864	EPI_ISL_125921	A/wild_bird/Korea/A01/2011
EPI387869	EPI_ISL_125922	A/wild_bird/Korea/A13/2010
EPI387866	EPI_ISL_125926	A/mallard/Korea/1203/2010
EPI256770	EPI_ISL_74722	A/muscovy_duck/Thailand/CU-LM4754/2009
EPI161537	EPI_ISL_391	A/duck/Hokkaido/24/04
EPI161527	EPI_ISL_16180	A/duck/Hokkaido/W87/2007
EPI314770	EPI_ISL_89365	A/duck/Thailand/LM-CU4777/2009
EPI600858	EPI_ISL_182001	A/duck/Jiangxi/4759/2009
EPI296592	EPI_ISL_84567	A/Eurasian_wigeon/Netherlands/4/2007
EPI889890	EPI_ISL_243520	A/mallard_duck/Netherlands/16/2006
EPI169461	EPI_ISL_26345	A/Fowl/Hampshire/PD378/1985
EPI3453372	EPI_ISL_19281517	A/Chicken/Netherlands/24010003-006010/2024
EPI_ISL_19750620	EPI4019318	A/Guangxi/01591/2024
EPI_ISL_162076	EPI530534	A/Jiangxi-Donghu/346-2/2013(H10N8)
EPI1868394	EPI_ISL_2380100	A/Jiangsu/428/2021
EPI497477	EPI_ISL_152846	A/Jiangxi-Donghu/346/2013
EPI1868385	EPI_ISL_2379892	A/Jiangsu/428/2021(H10N3)
EPI2970696	EPI_ISL_18846022	A/Zhejiang/CNIC-ZJU01/2023
EPI530526	EPI_ISL_162075	A/Jiangxi-Donghu/346-1/2013
EPI572713	EPI_ISL_174265	A/Jiangxi/1/2013
EPI572720	EPI_ISL_174265	A/Jiangxi/1/2013
EPI3227438	EPI_ISL_19067870	A/Yunnan/0110/2024
