# Supplementary figures and images for: Divergent Avian Influenza H10 Viruses from Sympatric Waterbird Species in Italy: Zoonotic Potential Assessment by Molecular Markers
Source: Microorganisms. 2025 Nov 12;13(11):2575. doi: 10.3390/microorganisms13112575 (PMC12654176; doi:10.3390/microorganisms13112575)

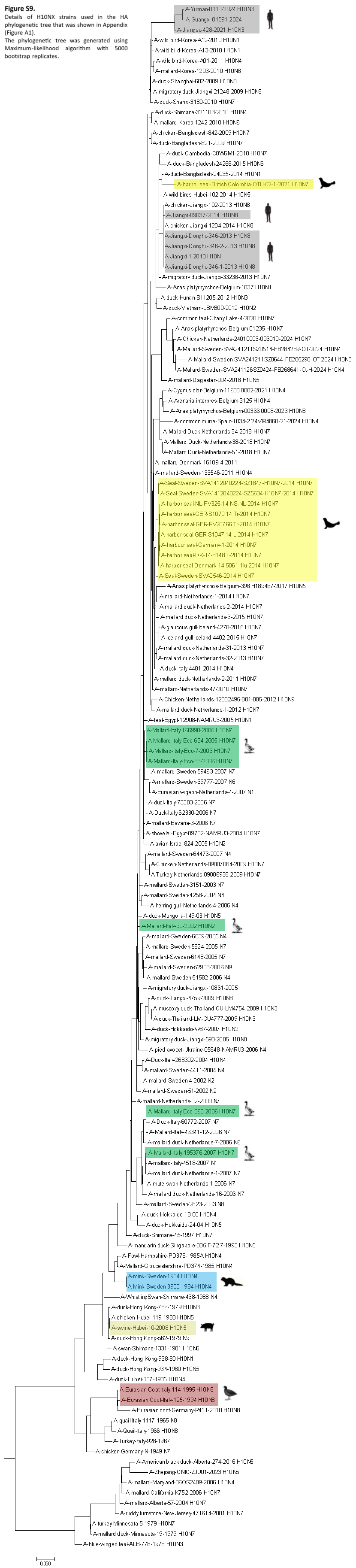

Supplement: Supplementary file 1 [file microorganisms-13-02575-s001.zip › Figure S9.PNG]
